# A bibliometric analysis and systematic review of drug repurposing against drug-resistant ESKAPE pathogens: a particular focus on *Pseudomonas aeruginosa*

**DOI:** 10.3389/fmicb.2025.1669585

**Published:** 2025-10-16

**Authors:** Sitong Guo, Lin Li, Qianqian Zhang, Huanxiang Liu, Xiaojun Yao, Liang Liang, Chunxia Chen

**Affiliations:** 1 Department of Pharmacy, The People’s Hospital of Guangxi Zhuang Autonomous Region, Guangxi Academy of Medical Sciences, Nanning, Guangxi, China; 2 Faculty of Applied Sciences, Macao Polytechnic University, Macao, Macao SAR, China; 3 Department of Laboratory Medicine, The People’s Hospital of Guangxi Zhuang Autonomous Region, Guangxi Academy of Medical Sciences, Nanning, Guangxi, China

**Keywords:** drug repurposing, drug-resistant ESKAPE, drug-resistant *Pseudomonas aeruginosa*, bibliometric analysis, systematic review

## Abstract

**Purpose:**

Drug-resistant ESKAPE pathogens represent a major global health challenge. This study included a comprehensive bibliometric analysis and systematic review to evaluate drug repurposing efforts against these pathogens, with a particular focus on *Pseudomonas aeruginosa*.

**Methods:**

We searched the Web of Science Core Collection (2001–April 2025) using the query “ESKAPE AND Drug Resistance AND Drug Repositioning” and performed bibliometric analysis with Bibliometrix (RStudio 4.3.2), VOSviewer 1.6.20, and CiteSpace 6.2R6. In parallel, a systematic review was conducted across PubMed, Embase, Web of Science, and Cochrane Library to identify non-antibiotic agents with reported activity against resistant ESKAPE pathogens.

**Results:**

A total of 443 articles were analyzed bibliometrically, and 141 eligible studies were included in the systematic review, among which 31 focused on drug-resistant *P. aeruginosa*. The United States, China, and India were the leading contributors, with notable institutional collaborations. Repurposed agents such as niclosamide and mitomycin C exhibited antibacterial activity through mechanisms including membrane permeability disruption, quorum sensing inhibition, and biofilm suppression. Many agents also showed synergistic effects when combined with conventional antibiotics.

**Conclusion:**

By integrating bibliometric mapping with systematic evidence synthesis, this study uniquely highlights both research trends and therapeutic potential in drug repurposing for ESKAPE pathogens. While repurposing offers advantages of reduced cost and faster development, translation remains constrained by toxicity, pharmacokinetics, and regulatory hurdles. Limitations include restriction to English-language studies and the use of selected databases. Future efforts should emphasize *in vivo* validation, clinical trials, and innovative delivery systems to accelerate clinical application.

**Systematic review registration:**

https://www.crd.york.ac.uk/prospero/, identifier CRD420251053437.

## Introduction

Antimicrobial resistance (AMR) has been recognized as one of the leading causes of death worldwide. In 2019, an estimated 4.95 million deaths were associated with AMR, with 1.27 million deaths directly attributable to bacterial resistance (Antimicrobial Resistance Collaborators, 2022). Without intervention, AMR may cause up to 10 million deaths annually by 2050 (Antimicrobial Resistance Collaborators, 2022; Walsh et al., 2023). The World Health Organization (WHO) warns that rising resistance could render even minor infections fatal (World Health Organization [WHO], 2014). Among resistant organisms, the “ESKAPE” pathogens–*Enterococcus faecium*, *Staphylococcus aureus*, *Klebsiella pneumoniae*, *Acinetobacter baumannii*, *Pseudomonas aeruginosa*, and *Enterobacterales*–are most concerning (Wan et al., 2020). Listed as WHO priority pathogens in 2017 (De Oliveira et al., 2020), they drive a disproportionate share of global AMR disease burden, causing severe hospital infections, treatment failures, and increased mortality and costs (Santajit and Indrawattana, 2016; Peterson and Kaur, 2018). Their spread, aided by genetic mutations and mobile genetic elements, further amplifies their impact (Peterson and Kaur, 2018; Santajit and Indrawattana, 2016).

Drug repurposing (also termed repositioning, reprofiling, or re-tasking) offers a practical response to the urgent need for new antimicrobials. By using existing pharmacological and safety data, it can cut development costs by over $1 billion and halve FDA approval timelines compared with new drugs (Boyd et al., 2021; Low et al., 2020). Regulatory interest is growing: the EMA launched a repurposing pilot in 2021 (Jonker et al., 2024), and nearly 30% of recent FDA approvals involve repurposed agents (Ashburn and Thor, 2004). Its advantages include lower risk of unforeseen toxicity, fewer late-stage trial failures, and faster regulatory clearance (Sun et al., 2016a). Importantly, many repurposed drugs act via mechanisms distinct from traditional antibiotics, enabling them to bypass resistance pathways (Gontijo et al., 2021; Kaul et al., 2019). High-throughput screens have identified numerous candidates with unexpected antibacterial effects (Ayon, 2023; Blasco et al., 2024; Liu et al., 2021), and combining such agents with standard antibiotics may further enhance efficacy (Boyd et al., 2021).

Despite these advances, knowledge remains fragmented. Bibliometric analysis, integrated with systematic review, can reveal research trends, influential contributors, and thematic hotspots, linking scientific evidence to translational priorities (Agarwal et al., 2016; Grant and Booth, 2009; van Eck and Waltman, 2010). Yet most prior reviews lack such comprehensive visual mapping, and systematic evaluations of non-antibiotic agents–especially against *P. aeruginosa*–are still scarce (Aggarwal et al., 2024; Jampilek, 2022; Liu et al., 2021). This study addresses these gaps by combining bibliometric mapping and systematic review to provide a holistic overview of the field, highlight promising agents, and clarify mechanisms with translational relevance.

## Methods

### Search strategy

The literature was systematically reviewed using the PRISMA (Reporting Items for Systematic Reviews and Meta-Analyses) guideline. We searched PubMed, Embase, Web of Science, and Cochrane Library databases to identify studies using drug repurposing approaches against drug-resistant ESKAPE pathogens from inception to April 7, 2025. We used only published articles in the English language. The search strategy was: (“*Enterobacterales*” OR “*Acinetobacter baumannii*” OR “*Pseudomonas aeruginosa*” OR “*Klebsiella pneumoniae*” OR “*Staphylococcus aureus*” OR “*Enterococcus faecium*”) AND “Drug Resistance” AND “Drug Repositioning.” Subject headings and related keywords were employed to find potential articles. The search strategy is displayed in Supplementary Table 1. The references of relevant studies were inspected to identify extra and follow-up studies. Two authors independently carried out database searches, and they excluded duplicates, examined titles and abstracts, and comprehensively evaluated the full texts to select potentially suitable studies. A third author joined the discussion and resolution when there were any discrepancies. The protocol of review has been registered in PROSPERO (CRD420251053437).

### Bibliometric analysis

As one of the most extensively utilized academic databases, the Web of Science (WoS) encompasses over 12,000 high-quality journals and maintains comprehensive citation records (Jiang et al., 2023). We selected the Web of Science Core Collection (WoSCC) as it is widely regarded as the most authoritative and standardized source for bibliometric research, ensuring reproducibility when applying tools such as CiteSpace, VOSviewer, and Bibliometrix. If databases such as Scopus and Dimensions were added, their high degree of overlap with WoSCC would lead to redundancy, and WoSCC provides a more consistent dataset, particularly suitable for citation-based analysis. We conducted a search and export of relevant articles from the WoSCC, utilizing all available database versions. To ensure the relevance and quality of our dataset, we limited the search results to articles and article reviews. We then carefully selected the relevant publications and saved them in a plain.txt format for further analysis (Pilkington, 2018).

The software tools utilized for bibliometric analysis include Bibliometrix R package (R Core Team, 2016), VOSviewer 6.2R6 (van Eck and Waltman, 2010), and CiteSpace 6.2R6 (Synnestvedt et al., 2005). The Bibliometrix R package primarily focuses on quantitative analysis, where authors are extracted from the “AU” field within the dataset, the publication year is extracted from the PY field, keywords are extracted from the DE field, and the number of citations is extracted from the TC field. In this review, the Bibliometrix software (version 4.0.0) was used to tally the quantity of publications along with their citations, assess the frequency of key terms, gauge the intensity of cooperation among countries/authors, and develop a three-field plot visualization for keyword concurrence analysis.

**FIGURE 1:**
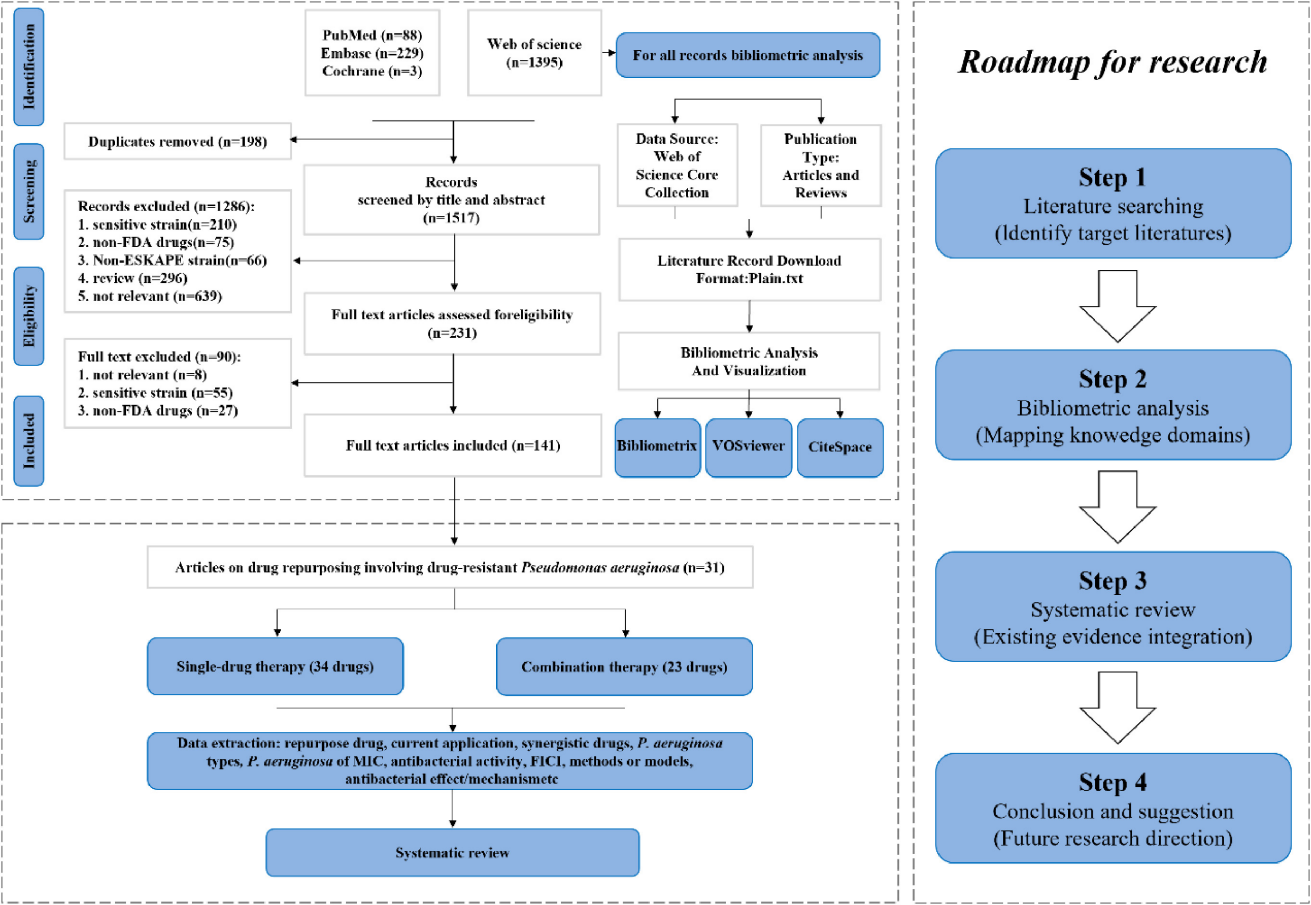
Flowchart of study selection and overview of study methodology.

A comprehensive overview of the bibliometric analysis systematic review and process was presented in Figure 1, providing a clear understanding of the methodology utilized in the analysis.

### Data extraction

After the final selection of the articles to be included in the study, the required data was extracted onto a customized sheet. Three reviewers independently extracted the corresponding data from each article, including the repurpose drug, current application, Synergistic drugs, *P. aeruginosa* types, *P. aeruginosa* of Minimum inhibitory concentration (MIC), antibacterial activity, Fractional Inhibitory Concentration Index (FICI), methods or models, antibacterial effect/mechanism, anti-biofilm activity, anti- quorum sensing (anti-QS) or anti-virulence, country of study and year of publication.

## Result

### Literature publication and citations

Initially, 1,715 records from electronic databases were screened through a comprehensive search. After removing 198 duplicates and screening the titles and abstracts of the remaining records, 1286 studies were excluded. After full-text reading and evaluation, ultimately 90 studies met the inclusion criteria, among which 31 studies involved drug repositioning of drug-resistant *P. aeruginosa* (Supplementary Table 2). A total of 1,395 articles related to drug repositioning of drug-resistant ESKAPE pathogens were retrieved from the Web of Science database, among which 449 were included in the Web of Science Core Collection. By restricting language type, time and research type, 443 articles were finally included for quantitative analysis, including 376 articles and 67 reviews.

**FIGURE 2:**
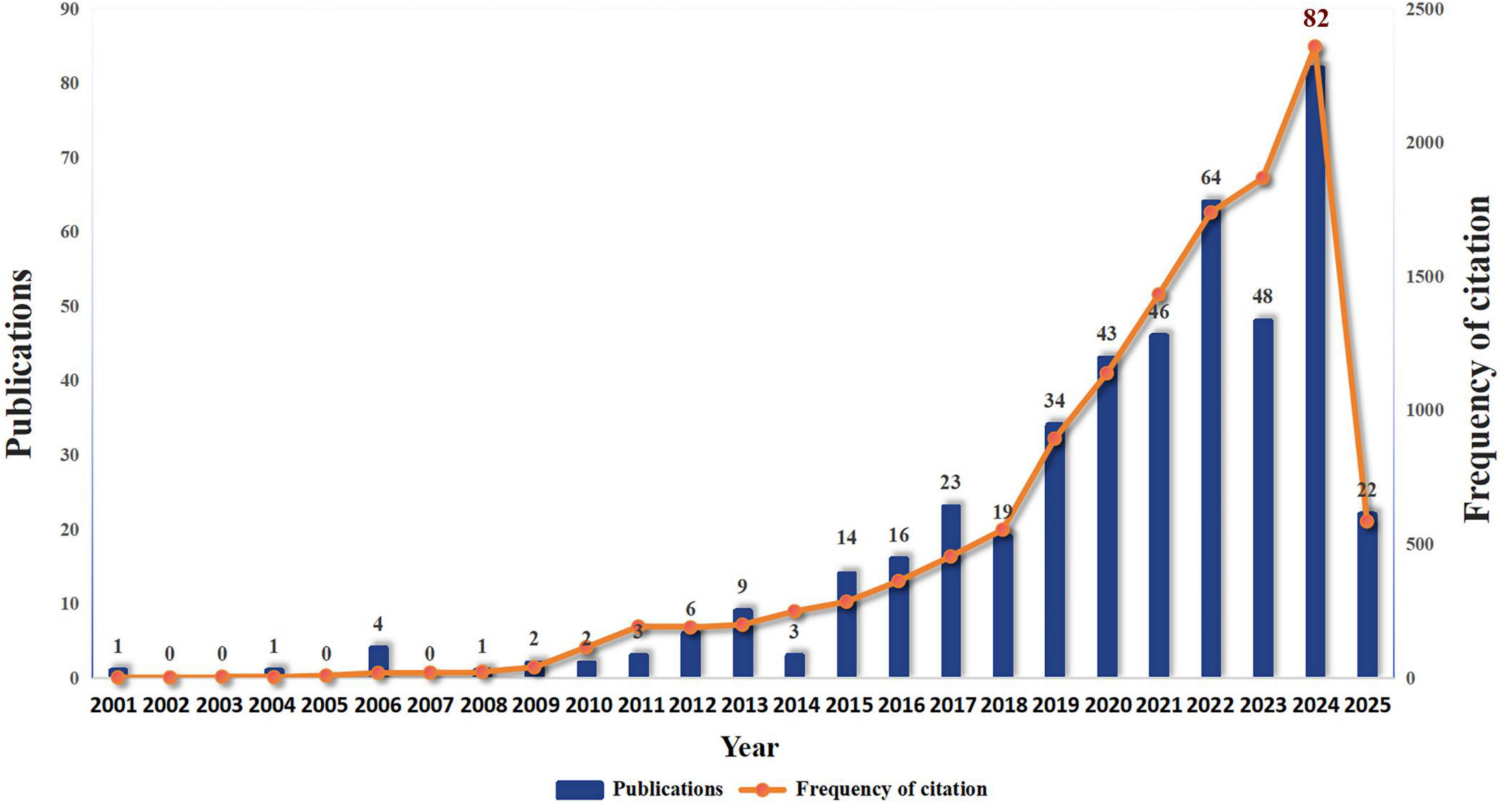
Annual publication volume in the field of drug repositioning of drug-resistant ESKAPE pathogens.

As shown in Figure 2, the annual publication volume and citation volume showed an overall fluctuating upward trend. Since 2008, the growth rate of publication volume has increased year by year, reaching a peak in 2024 (82 articles), indicating that the research activity in the field is continuing to rise. Since only data before April in 2025 are counted, the publication volume data for 2025 is not sufficient for the time being.

### Research countries/regions

**FIGURE 3:**
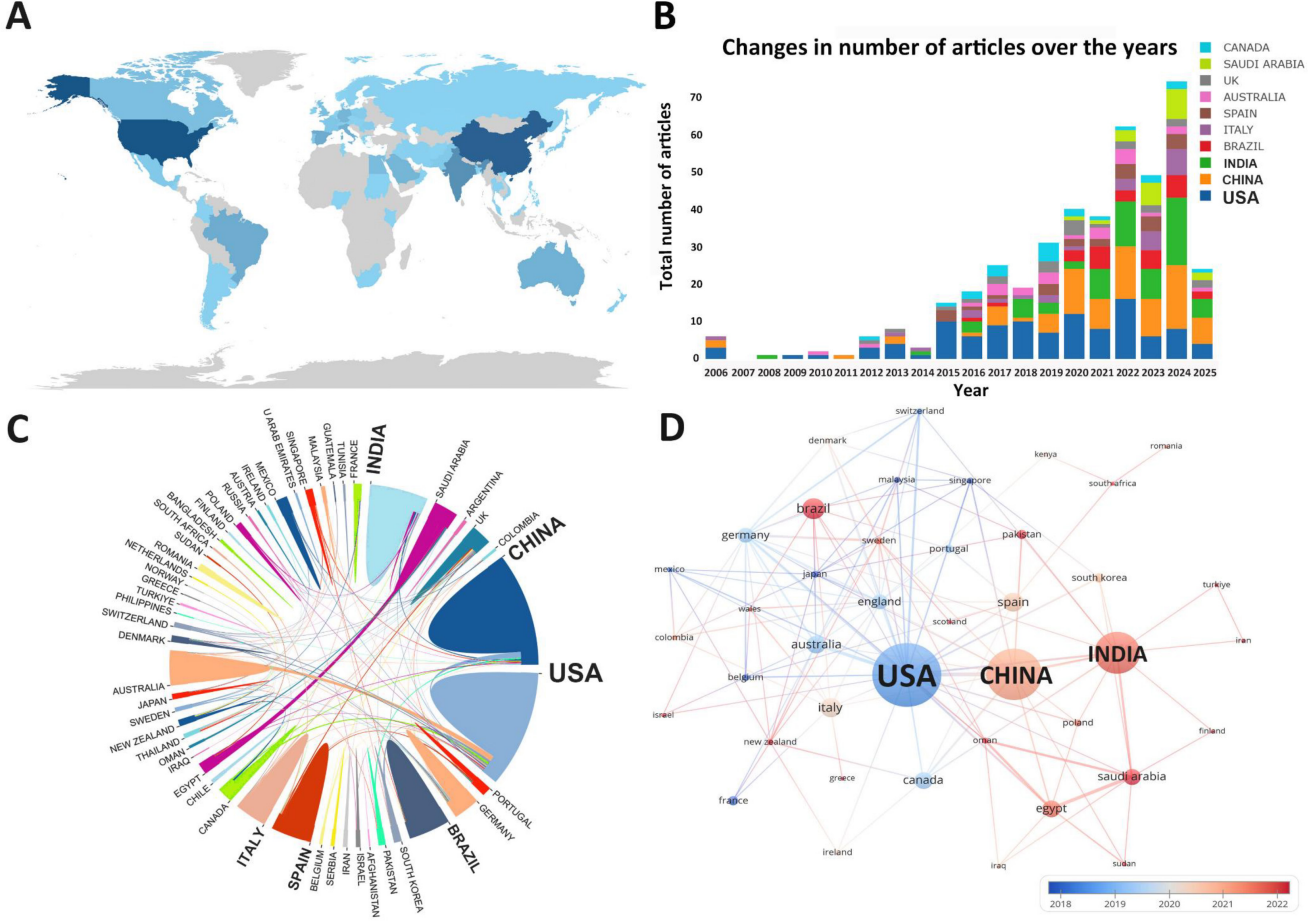
Country/region analysis. **(A)** Geographical distribution of research countries; **(B)** annual publication volume trend of the top 10 countries/regions from 2001 to 2025; **(C)** visualization of countries/regions for international cooperation; **(D)** time network of country/region cooperation. The thickness of the line reflects the intensity of the number of collaborations, and the color mapping is based on the average year of collaboration, from dark blue to yellow, representing the earliest to the most recent collaboration.

The research on drug repositioning for drug-resistant ESKAPE pathogens covers 62 countries/regions around the world. The top 10 countries with the most publications were statistically analyzed (Supplementary Table 3). The United States ranked first with 110 publications and 5,740 citations, followed by China (84,1959) and India (65,943), indicating that the United States has made great contributions and had a positive impact in this field. We visualized the publication and cooperation of countries/regions. The geographical distribution map in Figure 3A shows that the papers in the field of drug repositioning for drug-resistant ESKAPE pathogens are mainly published in Asian and North American countries. Figure 3B shows the annual publication volume of the top ten countries from 2006 to 2025. It can be observed that China and India have the highest annual growth rate. Figure 3C shows the level of international collaboration. The single-country author ratio (SCP) indicates the number of papers whose authors are from the same country, and the international collaboration ratio (MCP) indicates the number of papers that are co-authored with authors from other countries. The MCP ratio indicates the ratio of international collaboration. Combined with the chart, Australia (68.5%), Egypt (58.8%), and South Korea (25%) have relatively high rates of international collaboration. Figure 3D shows the average time for 43 countries to engage in international cooperation when the threshold is 2. Total link strength (TLS) indicates the intensity of cooperation. The United States is the first country to start international cooperation and has the most frequent cooperation with other countries (TLS = 69), while Turkey is the country that started international cooperation most recently.

### Institutional contribution

**FIGURE 4:**
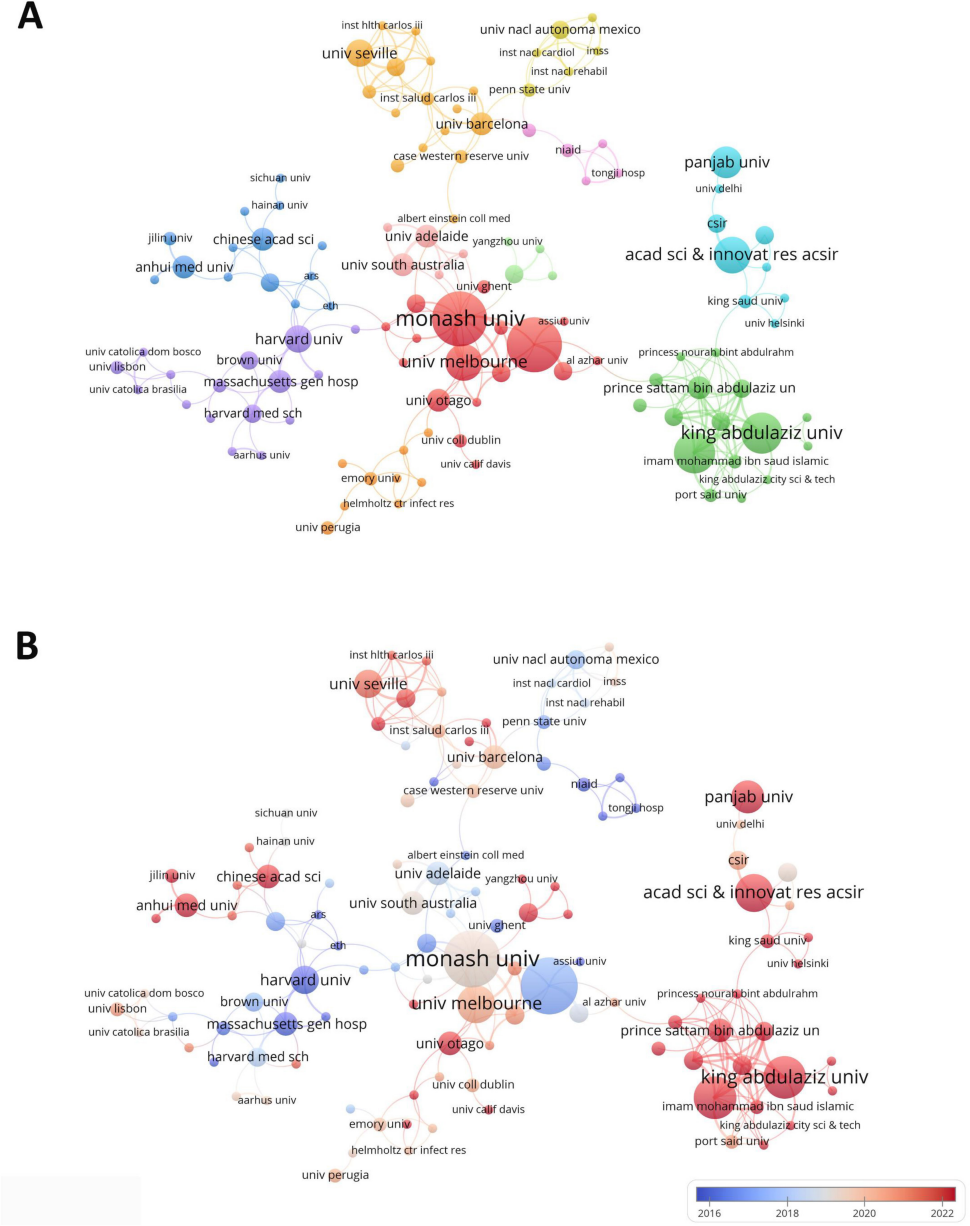
Institutional collaboration diagram. **(A)** Institutional collaboration co-occurrence map; **(B)** institutional collaboration timeline map. The thickness of the line reflects the intensity of the number of collaborations, and the color mapping is based on the average year of collaboration, from dark blue to dark red, representing the earliest to the most recent collaboration.

A total of 807 institutions were involved in research on drug repositioning for drug-resistant ESKAPE pathogens A statistical analysis was conducted on the top 10 institutions with the highest number of publications (Supplementary Table 4). The institutions engaged in related scientific research are mainly from the United States, Australia and India. The first-ranked institution is Purdue University from the United States (12 publications, 797 citations), followed by Monash University in Australia (12 publications, 556 citations), and King Abdulaziz University in Saudi Arabia (9 publications, 149 citations). Figure 4A shows the institutional cooperation relationship. Monash University (TLS = 29) has the most frequent and close cooperation with other institutions, while Purdue University (TLS = 13) and Monash University (TLS = 29) have formed the largest cooperation network with the two as the main body, which has promoted the development of this field. Institutions in the United States were the first to carry out academic research and cooperation in this field (Figure 4B), indicating that the United States has established a relatively complete institutional collaboration system, which has a far-reaching impact on the contribution to this field.

### Author analysis

**FIGURE 5:**
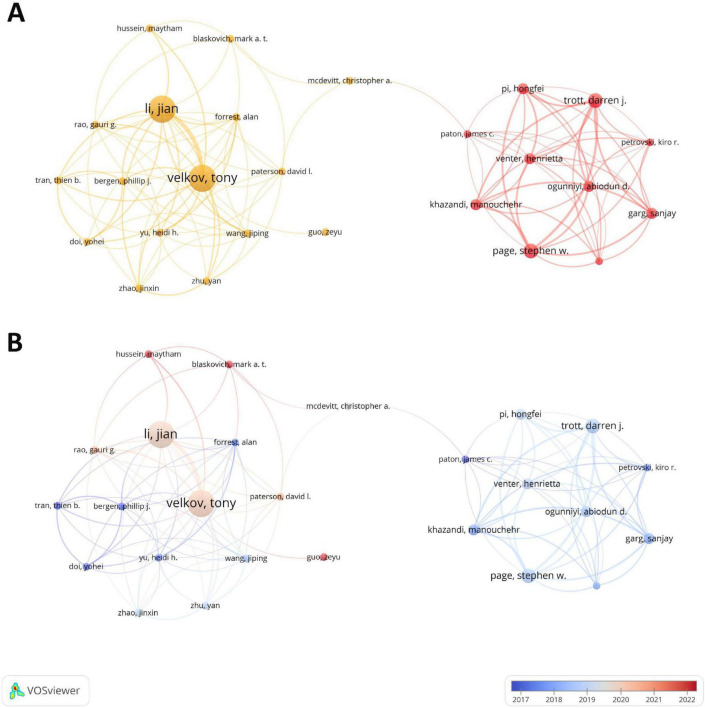
Author collaboration diagram. **(A)** Author collaboration co-occurrence map; **(B)** author collaboration time network. The thickness of the line reflects the intensity of the number of collaborations; the color mapping is based on the average year of collaboration, from dark blue to dark red, representing the earliest to the most recent collaborations.

A total of 2,570 authors have contributed to the research in the field of drug repositioning of drug-resistant ESKAPE pathogens. Supplementary Table 5 lists the top ten authors who published related papers. The first place is Li, Jian (8 papers, 388 citations) and Velkov, Tony (8 papers, 383 citations), and the third place is Page, Stephen and Trott, Darren (4 papers, 77 citations). The H-index is a quantitative indicator used to evaluate the academic influence of researchers. Among them, Li, Jian has the highest H-index, with a value of 8. A visual analysis of the author cooperation team is performed. Figure 5 uses a threshold of 2 to include 301 authors, of which only 26 authors have cooperation and contact. The results showed that two cooperation networks were formed among the authors, namely the team led by Li. Jian, Velkov, and Tony and the team led by Trott, Darren, Page, and Stephen, showing the characteristics of highly close teamwork. Among them, the team led by Li. Jian, Velkov, and Tony is still active in this field, and has made important contributions to the field of drug repositioning for drug-resistant ESKAPE pathogens, and has a certain authority.

### Highly productive journals and influential articles

**FIGURE 6:**
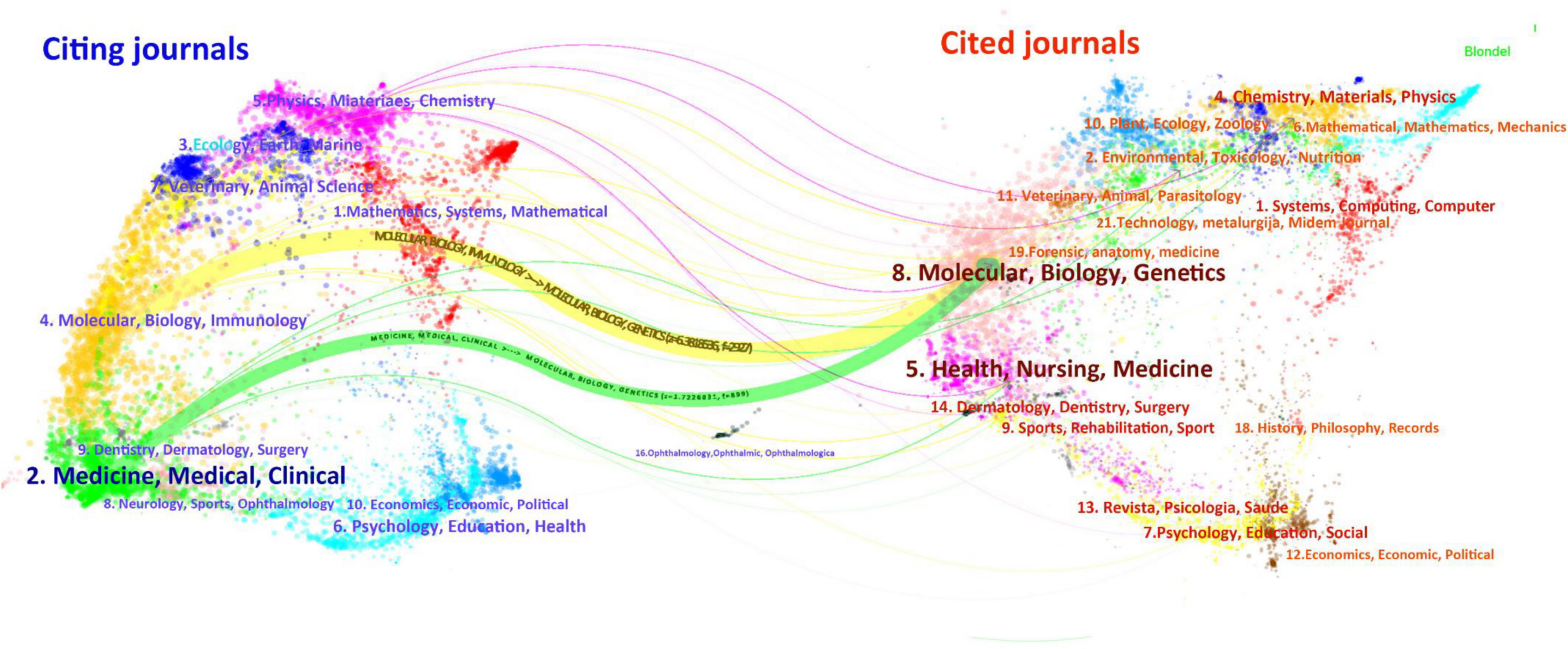
Journal double-image overlay analysis.

Supplementary Table 6 presents the ten journals with the highest number of publications on drug repositioning for drug-resistant ESKAPE pathogens. Frontiers in Microbiology topped the list with 30 articles, Antibiotics-Basel ranked second with 25 articles, and Antimicrobial Agents and Chemotherapy ranked third with 18 articles. The top 10 journals in terms of JCR classification are mainly in the Q1 zone, and the research topics of the journals are related to microbiology and anti-infection. The most influential journal is the International Journal of Antimicrobial Agents, with an Impact Factor (IF) of 4.6. In order to visualize the relationship between journal citations and citations and understand the subjective distribution and development patterns of disciplines, we conducted a double-graph overlay analysis of journals, as shown in Figure 6. The left side is the citing journals and the right side is the cited journals. The colored paths between the two sides reflect the reference connection. There are mainly one green and one yellow path in the figure, indicating that Molecular, Biology, Immunology and Medicine, Medical, Clinical often cite journals in the disciplines of Molecular, Biology, Genetics.

**FIGURE 7:**
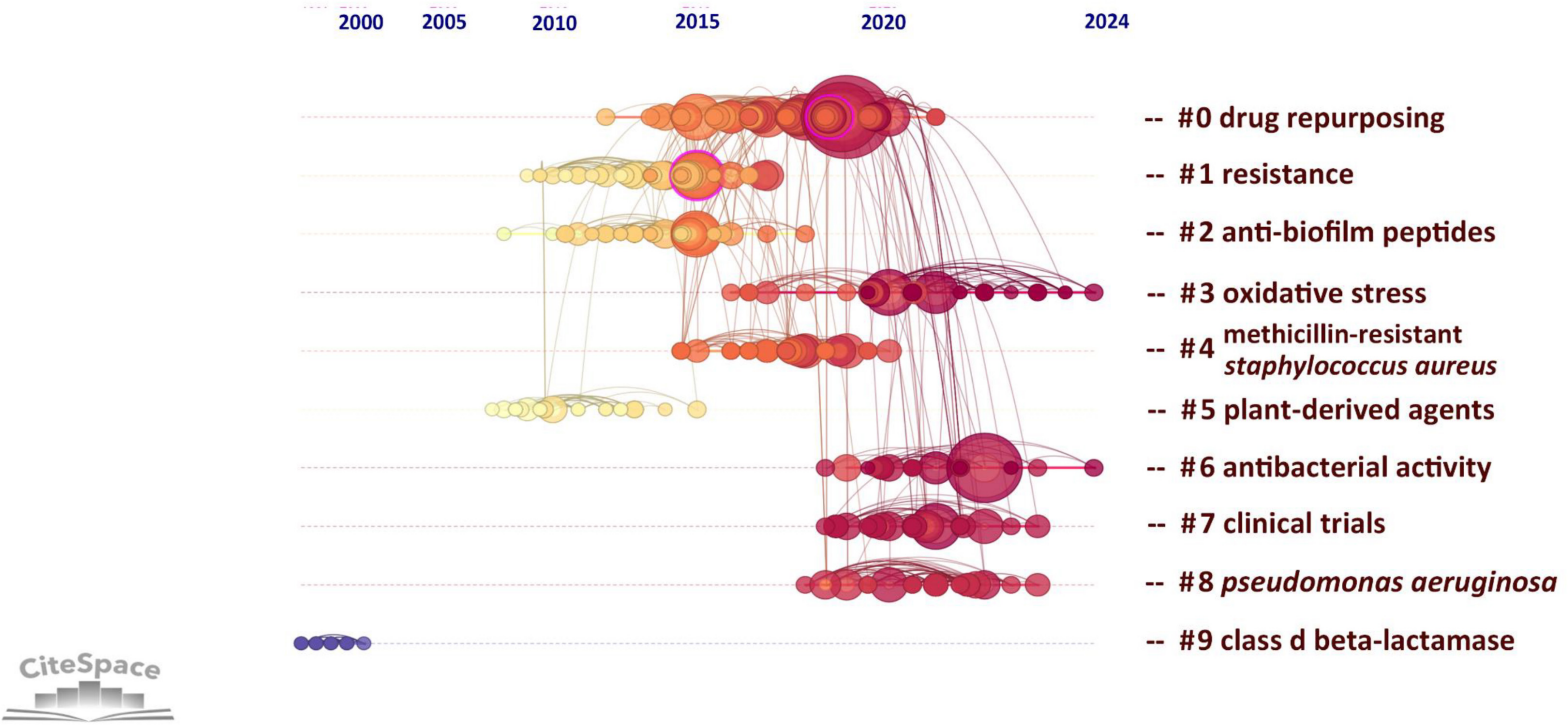
Timeline of research literature on drug repositioning of drug-resistant ESKAPE. A cluster timeline reflects a research topic; from left to right corresponds to the past to the present; the size of the node reflects the frequency of occurrence with the research topic.

Supplementary Table 7 highlights the ten studies on drug repositioning for drug-resistant ESKAPE pathogens that have attracted the greatest interest among researchers. These studies are mainly published in natural science and microbiology journals. The results of these studies have greatly influenced the development of the field and the research direction of scholars. Among them, the most frequently cited document is Antibiotics for Emerging Pathogens published by Fischbach, MA in the journal Science in 2009. The article points out that drug-resistant pathogens are becoming increasingly common, but the development of new antibiotics is progressing slowly. The article introduces methods for finding new “scaffolds” by mining microbial niches to obtain natural products and reusing synthetic molecular libraries. It also explores new directions for target-based antibiotic discovery strategies, emphasizing that new “scaffolds” are the key to dealing with drug resistance, and new scaffolds with different characteristics may play a role in future treatments. It ranks first with an average of 83 citations per year and a total of 1,421 citations. Through the analysis of co-citations of literature, we can reveal the research hotspots in the current discipline. Figure 7 analyzes the co-citations in the form of a timeline, which can show the changes in research hotspots over time. The results form 10 clusters, of which the largest cluster is “drug repurposing” (#0). The earliest research topic in this field was “Class-D β-lactamase” (#9). The current research hotspots are mainly concentrated on “drug repurposing,” “antibacterial activity” and “oxidative stress,” indicating that drug repurposing has received widespread attention in the treatment of ESKAPE infection, mainly focusing on the antibacterial activity and mechanism of repositioned drugs.

### Research hotspot analysis

**FIGURE 8:**
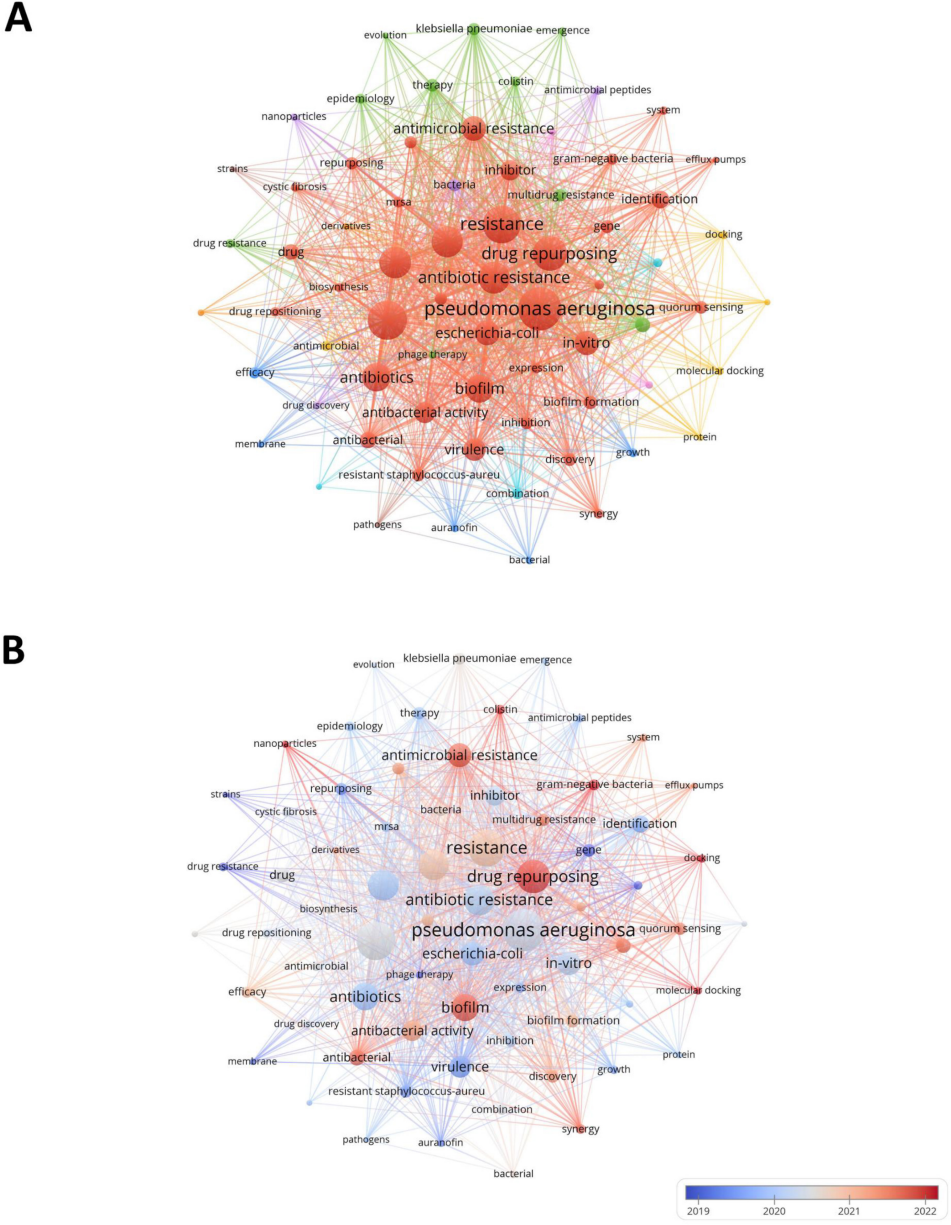
Keyword co-occurrence map. **(A)** Keyword co-occurrence map; **(B)** keyword time network map.

Through keyword analysis, we can identify the hot areas of current academic research and reveal the evolution of hot topics, so as to grasp the research dynamics, trends, research topics and potential interdisciplinary connections in the field of drug repositioning of drug-resistant ESKAPE pathogens, and establish the framework of the drug repositioning field of drug-resistant ESKAPE pathogens. The threshold was set to 10 and 70 of the 2,232 keywords retrieved from the abstract and title met the threshold. The keyword co-occurrence map was drawn using VOSviewer software. One node represents one keyword, and the node size is positively correlated with the frequency of keyword occurrence. The connections between nodes form different clusters, which are distinguished by different colors. The distance between nodes shows the connection strength. As shown in Figure 8A, the 70 keywords are divided into 9 clusters, focusing on different research topics: (1) The red and brown clusters focus on drug-resistant ESKAPE pathogens and their characteristics research and drug discovery. Such as *P. aeruginosa*, *Staphylococcus aureus*, resistance, drug repurposing, etc.; (2) Green and pink clusters focus on the epidemiology, treatment and antimicrobial research of drug-resistant bacteria, such as *Acinetobacter Baumannii*, epidemiology, phage therapy, etc., which jointly involve the epidemiological characteristics of drug-resistant bacteria, the exploration of treatment methods and the research of related antimicrobial drugs; (3) Blue, light blue and orange clusters revolve around drug-related research. Examples include auranofin and ciprofloxacin, encompassing various aspects such as drug efficacy, pharmacokinetics, the characteristics of different drugs, combination therapy effects, and pharmacokinetic studies; (4) Yellow and purple clusters focus on research methods and new antimicrobial methods. For example, it combines molecular docking, protein research methods with antimicrobial peptides and nanoparticles, involving the exploration of research techniques at the molecular level to new antimicrobial materials and drug discovery pathways.

In Figure 8B, the average publication year of each keyword is used as the score for color mapping, illustrating the temporal distribution of keyword clusters. Early research mainly focused on the basic characteristics of ESKAPE pathogens. The exploration was carried out around the essential characteristics of ESKAPE pathogens, such as studying strains and growth, trying to find ways to deal with drug resistance from these typical strains. Recent research has focused more on mechanisms and emerging technology applications, such as multidrug resistance, antimicrobial resistance, and drug repurposing, showing that researchers have explored more evidence for drug reuse through in-depth analysis of drug resistance mechanisms. In addition, it also focuses on hot spots such as nanoparticles and molecular docking, reflecting the trend and application exploration of accurately screening drugs with the help of molecular simulation technology in drug repositioning research, which provides new ways to solve the problem of drug resistance.

**FIGURE 9:**
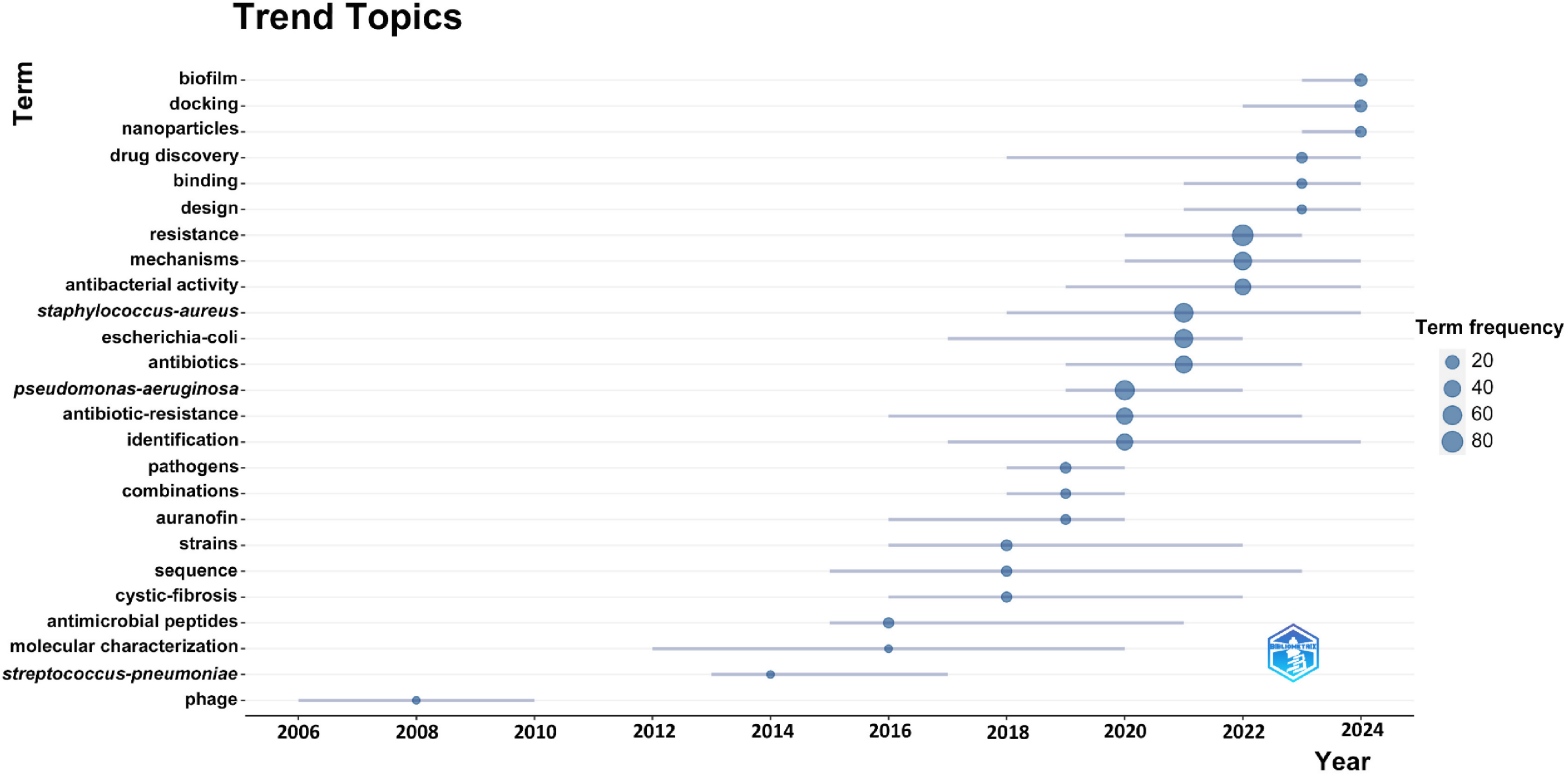
Topic trend map.

We use keyword emergence analysis to identify keywords that emerge frequently in a short period of time, which can more accurately capture the new scientific research directions and research theme changes in the field of drug repositioning for drug-resistant ESKAPE pathogens. This article uses the bibliometrix package to display the keyword emergence results in the form of pictures (Figure 9). It can be found that early research was mainly related to the study and treatment of the characteristics of ESKAPE pathogens, and understanding their basic properties as pathogens, which clarified the target objects for drug repositioning. When traditional antibiotics fail, they turn to non-antibacterial drug treatment methods, such as studying biotherapeutic phages and auranofins; and recent research is more closely centered on drug repositioning in the treatment of MDR ESKAPE pathogens, and in-depth research on the resistance mechanism of drug-resistant bacteria, precision drug screening, and the application of new drug carriers, such as biofilm, molecular docking of therapeutic methods, nanoparticles, and other new technologies. The research time span reflects the continuity of the topic, with “molecular characterization” and “gene sequencing” being the longest-standing areas of focus. Keyword frequency highlights the popularity of research topics, with “resistance” and “*P. aeruginosa*” emerging as the most frequently studied in the field of drug repositioning for drug-resistant ESKAPE pathogens.

### Drug repurposing against drug-resistant *P. aeruginosa*

#### Single-drug therapy

A wide range of FDA-approved non-antibacterial drugs have demonstrated direct antibacterial activity against MDR or XDR *P. aeruginosa in vitro*, including anticancer, cardiovascular, antiparasitic, non-steroidal anti-inflammatory (NSAIDs), and psychiatric drugs. Among them, anticancer agents such as mitomycin C (MIC 0.031–0.062 μg/mL) (Domalaon et al., 2019a; Svedholm et al., 2024; Wu et al., 2020), carmofur (0.0125–0.2 mM) (Di Bonaventura et al., 2023), and doxorubicin (2–32 μg/mL) (Domalaon et al., 2019a) exhibited strong activity, largely through DNA damage and cell membrane disruption. The cardiovascular drug amlodipine (25–50 μg/mL) not only inhibited bacterial growth but also significantly attenuated biofilm formation and virulence factor expression (Sharma et al., 2024a,b). Among antiparasitic agents, niclosamide (2 μg/mL) showed the lowest MIC, likely related to disruption of the proton motive force, whereas oxirachamide and broxiquinolone showed moderate activity (Di Bonaventura et al., 2023; Domalaon et al., 2019c). Certain NSAIDs (e.g., indomethacin, 34.2 μg/mL) and psychiatric drugs (e.g., sertraline, 16 μg/mL) displayed moderate inhibitory effects, with additional quorum sensing (QS) and biofilm interference activities (Endo et al., 2025; Serafin et al., 2020; Sharma et al., 2024b). Other agents, such as vitamin C, exhibited auxiliary anti-bacterial or anti-virulence effects. Collectively, these findings suggest that multiple non-antibacterial drugs possess repurposing potential against *P. aeruginosa* through multi-target mechanisms, including enhanced membrane permeability, metabolic interference, and virulence suppression. Among them, mitomycin C, niclosamide, and amlodipine showed the most consistent and potent activity. Research in this area has expanded globally since 2015, with contributions primarily from China, the United States, Canada, Brazil, India, and Australia (Table 1).

**TABLE 1:** Drug repurposing against drug-resistant *P. aeruginosa*.

No.	Repurpose drug (current application)	PA types	PA of MIC (μg/ml)	Antibacterial activity	Methods or models	Antibacterial effect/ mechanism	Anti-biofilm activity	Anti-QS or anti-virulence	Country of study	Year	Refs
1	5-fluorouracil (anticancer)	MDR	64	NR	*In vitro*	NR	NR	NR	Canada	2019	Di Bonaventura et al., 2023; Domalaon et al., 2019a; Sharma et al., 2024b
		MDR	0.0125–0.05 mM	DR-*S. aureus*	*In vitro*	NR	Preventing biofilm formation (*P. aeruginosa*)	NR	Italy	2023	
		MDR	8.60	MDR-*P. stutzeri*, MDR- *P. fluroscence*, MDR-*P. ptuida*	*In vitro*; molecular docking	NR	NR	NR	India	2024	
2	9-aminoacridine (HIV-1 inhibitor)	XDR	64	MRSA, XDR-*K. pneumoniae*, XDR-*A. baumannii*, XDR-*E. coli*, MDR-*E. faecium*	*In vitro*; acute wound infection mice model (*K. pneumoniae*)	Interacted with bacterial DNA and disrupted the proton motive force in *K. pneumoniae*	NR	NR	China	2023	She et al., 2023
3	Amlodipine (antihypertensive)	MDR	25–50	DR-*P. stutzeri*; DR-*P. fluorescens*; DR-*P. putida*	*In vitro*	Damage of the structural integrity (*P. aeruginosa*)	Reducing biofilm (*P. aeruginosa*)	Reducing the various virulence factors, including swimming and twitching motility, rhamnolipid, pyocyanin, and oxidative stress resistance against (*P. aeruginosa*)	India	2024	Sharma et al., 2024a,b
		MDR	9.62	MDR-*P. stutzeri*, MDR- *P. fluroscence*, MDR-*P. ptuida*	*In vitro*; molecular docking	NR	NR	NR	India	2024	
4	Ascorbic acid (vitamin)	MDR	30.65	MDR-*P. stutzeri*, MDR- *P. fluroscence*, MDR-*P. ptuida*	*In vitro*; molecular docking	NR	NR	NR	India	2024	Sharma et al., 2024b
5	Atorvastatin (antihyperlipidemic)	MDR	200.01	MDR-*P. stutzeri*, MDR- *P. fluroscence*, MDR-*P. ptuida*	*In vitro*; molecular docking	NR	NR	NR	India	2024	Sharma et al., 2024b
6	Auranofin (antirheumatics)	MDR	0.0125–0.2 mM	NR	*In vitro*	NR	NR	NR	Italy	2023	Di Bonaventura et al., 2023
7	Broxyquinoline (antiparasitic)	MDR	0.2 mM	DR-*S. aureus*, DR-*S. maltophilia*, DR-*B. cepacia*, DR-*A. baumannii*	*In vitro*	NR	NR	NR	Italy	2023	Di Bonaventura et al., 2023
8	Calaptin (antifungal)	MDR	56.70	MDR-*P. stutzeri*, MDR- *P. fluroscence*, MDR-*P. ptuida*	*In vitro*; molecular docking	NR	NR	NR	India	2024	Sharma et al., 2024b
9	Camptothecin (anticancer)	MDR	256–512	NR	*In vitro*	NR	NR	NR	Canada	2019	Domalaon et al., 2019a
10	Carmofur (anticancer)	MDR	0.0125–0.2 mM	DR-*S. aureus*, DR-*S. maltophilia*, DR-*B. cepacia*	*In vitro*	Damage cell membranes, with leakage and cytoplasm loss, by increasing membrane permeability (*P. aeruginosa*)	Preventing biofilm formation (*P. aeruginosa*)	NR	Italy	2023	Di Bonaventura et al., 2023
11	Celecoxib (non-steroidal antiinflammatory)	MDR	210	MDR-*P. stutzeri*, MDR- *P. fluroscence*, MDR-*P. ptuida*	*In vitro*; molecular docking	NR	NR	NR	India	2024	Sharma et al., 2024b
12	Ciclopirox (antifungal)	DR	10–30	DR/MDR-*E. coli*, DR/MDR-*K. pneumoniae*, DR/MDR-*A. baumannii*	*In vitro*	Ciclopirox affects galactose metabolism and LPS biosynthesis of *E. coli*	NR	Yes	USA	2013	Carlson-Banning et al., 2013; Zakaria et al., 2019
		MDR	8–64	NR	*In vitro*	Reduced pyocyanin production, protease secretion and biofilm formation (*P. aeruginosa*)	Decreased biofilm formation (*P. aeruginosa*)	Reduced pyocyanin production, decreased protease secretion in 46% isolates, lowered twitching and swarming motility (*P. aeruginosa*)	Egypt	2019	
13	Cisplatin (anticancer)	MDR	128	NR	*In vitro*	NR	NR	NR	Canada	2019	Domalaon et al., 2019a
14	Diclofenac (non-steroidal antiinflammatory)	MDR	85.70	MDR-*P. stutzeri*, MDR- *P. fluroscence*, MDR-*P. ptuida*	*In vitro*; molecular docking	NR	NR	NR	India	2024	Sharma et al., 2024b
15	Doxorubicin (anticancer)	MDR	2–32	NR	*In vitro*	NR	NR	NR	Canada	2019	Domalaon et al., 2019a
16	Ebselen (anticancer)	MDR	0.1–0.2 mM	DR-*S. aureus*, DR-*B. cepacia*	*In vitro*	Damage cell membranes, with leakage and cytoplasm loss, by increasing membrane permeability (*P. aeruginosa*)	NR	NR	Italy	2023	Di Bonaventura et al., 2023
17	Etoposide (anticancer)	MDR	16–128	NR	*In vitro*	NR	NR	NR	Canada	2019	Domalaon et al., 2019a
18	Hydroxychloroquine (antimalarial)	MDR	65.43	MDR-*P. stutzeri*, MDR- *P. fluroscence*, MDR-*P. ptuida*	*In vitro*; molecular docking	NR	NR	NR	India	2024	Sharma et al., 2024b
19	Indomethacin (non-steroidal antiinflammatory)	MDR	34.23	MDR-*P. stutzeri*, MDR- *P. fluroscence*, MDR-*P. ptuida*	*In vitro*; molecular docking	NR	NR	NR	India	2024	Sharma et al., 2024b
20	Mitomycin C (anticancer)	MDR	0.031–0.062	DR-*E. coli*, DR-*K. pneumoniae*, DR-*A. baumannii*, DR-*E. cloacae*	*In vitro*	NR	NR	NR	Canada	2019	Domalaon et al., 2019a; Svedholm et al., 2024; Wu et al., 2020
		MDR	0.125–8	NR	*In vitro*	NR	NR	NR	Sweden	2024	
		MDR	6–12 μM	MDR-*E. coli*, MDR-*E. cloacae*, MDR-*A. baumannii*, MDR-*K. pneumoniae*	*In vitro*	NR	NR	NR	China	2020	
21	Niclosamide (anthelmintic)	MDR	2	NR	*In vitro*	NR	NR	NR	Canada	2019	Domalaon et al., 2019c
22	Oxyclozanide (anthelmintic)	MDR	64	NR	*In vitro*	NR	NR	NR	Canada	2019	Domalaon et al., 2019c
23	Panobinostat (anticancer)	MDR	0.2 mM	DR-*S. aureus*	*In vitro*	NR	NR	NR	Italy	2023	Di Bonaventura et al., 2023
24	Paracetamol (antipyretic analgesic)	MDR	56.70	MDR-*P. stutzeri*, MDR- *P. fluroscence*, MDR-*P. ptuida*	*In vitro*; molecular docking	NR	NR	NR	India	2024	Sharma et al., 2024b
25	Paroxetine (antidepressants)	MDR	127.5	MRSA, MDR-*E. faecium*, XDR-*K. pneumoniae*, MDR- *A. baumannii*, MDR-*E. cloacae*, MDR-*E. coli*	*In vitro*	Oxidative stress induction, alterations in membrane permeability, and their capability to act as efflux pump inhibitors (*E. coli* or *S. aureus*)	NR	NR	Brazil	2025	Endo et al., 2025
26	Pentamidine (antimalarial)	MDR	16–512	NR	*In vitro*; infected *Galleria mellonella* larvae model (*P. aeruginosa*)	Substrate of the RND efflux pumps	NR	NR	Sweden	2024	Svedholm et al., 2024 Wu et al., 2020
	MDR	256 μM	MDR-*E. coli*, MDR-*A. baumannii*, MDR-*K. pneumoniae*	*In vitro*	NR	NR	NR	China	2020		
27	Promethazine (antihistamine)	MDR	60.4	MDR-*P. stutzeri*, MDR- *P. fluroscence*, MDR-*P. ptuida*	*In vitro*; molecular docking	NR	NR	NR	India	2024	Guedes et al., 2024; Sharma et al., 2024b
		DR	195.31–781.25	MDR-*S. aureus*, MDR- *E. faecalis*, MDR-*E. coli*	*In vitro*	NR	Reduced mature biofilm biomass (*P. aeruginosa*)	NR	Brazil	2024	
28	Pyridoxine (vitamin)	MDR	197	MDR-*P. stutzeri*, MDR- *P. fluroscence*, MDR-*P. ptuida*	*In vitro*; molecular docking	NR	NR	NR	India	2024	Sharma et al., 2024b
29	Rifampin (antituberculosis)	XDR	16–64	MDR/XDR-*E. coli*, MDR/XDR-*K. pneumoniae*, MDR/XDR-*A. baumannii*	*In vitro*	NR	NR	NR	China	2022	She et al., 2022
30	Sertraline (antidepressants)	MDR	16	MRSA, MDR-*S. haemolyticus*, MDR-*S. epidermidis*, MDR-*A. baumannii*	*In vitro*	NR	NR	NR	Brazil	2022	Endo et al., 2025; Serafin et al., 2020
		MDR	127.5	MRSA, MDR-*E. faecium*, XDR-*K. pneumoniae*, MDR- *A. baumannii*, MDR-*E. cloacae*, MDR-*E. coli*	*In vitro*	Oxidative stress induction, alterations in membrane permeability, and their capability to act as efflux pump inhibitors (*E. coli* or *S. aureus*)	NR	NR	Brazil	2025	
31	Sildenafil (idiopathic pulmonary arterial hypertension)	DR	3.12–6.25 mg/ml	NR	*In vitro*	NR	Inhibited and eradicated biofilms (*P. aeruginosa*)	NR	Brazil	2024	Barin et al., 2024
32	Sulforaphane (anticancer)	MDR	0.1 mM	NR	*In vitro*	NR	NR	NR	Italy	2023	Di Bonaventura et al., 2023
33	Tavaborole (antifungal)	MDR	0.1–0.2 mM	DR-*S. aureus*, DR-*S. maltophilia*, DR-*B. cepacia*, DR-*A. baumannii*	*In vitro*	NR	Dispersing preformed biofilms (*P. aeruginosa*)	NR	Italy	2023	Di Bonaventura et al., 2023
34	Tirapazamine (anticancer)	MDR	0.0125–0.2 mM	DR-*S. aureus*, DR-*B. cepacia*	*In vitro*	Damage cell membranes, with leakage and cytoplasm loss, by increasing membrane permeability (*P. aeruginosa*)	Dispersing preformed biofilms (*P. aeruginosa*)	NR	Italy	2023	Di Bonaventura et al., 2023

DR, drug resistance; MDR, multidrug resistance; MIC, minimum inhibitory concentration; NR, no reported; PA, *P. aeruginosa*; OS, quorum sensing; Refs, References; XDR, extensively drug resistant.

#### Combination therapy

Several non-antibacterial drugs also displayed significant synergistic effects when combined with traditional antibiotics, particularly polymyxins. For example, the anti-rheumatic agent auranofin and NSAIDs such as celecoxib reduced their MIC from >256 μg/mL to 0.5–4 μg/mL when combined with colistin, with FICI values < 0.5, indicating strong synergy (Feng et al., 2021; Sun et al., 2016b; Thangamani et al., 2015b). Antiparasitic drugs such as closantel, niclosamide, oxirane, and rafoxanide also showed marked synergy with colistin (FICI as low as 0.001), primarily via membrane damage and proton motive force disruption (Ding et al., 2024; Domalaon et al., 2019b, c; Lu et al., 2022). The α1-adrenergic antagonist doxazosin combined with ciprofloxacin suppressed QS, reduced virulence, and significantly diminished biofilm burden, while sildenafil in combination with colistin or cefepime effectively inhibited and eradicated *P. aeruginosa* biofilms (Elfaky et al., 2023). Antidepressants such as fluoxetine and chlorpheniramine enhanced the bactericidal activity of aminoglycosides or carbapenems by inhibiting efflux pumps, inducing ROS, and disrupting QS (Ahmed et al., 2024; de Melo Guedes et al., 2024). Mitomycin C, adenosine phosphate, and ribavirin also exhibited synergistic effects with antibiotics, simultaneously reducing MIC values and interfering with virulence regulatory networks (Domalaon et al., 2019a; Svedholm et al., 2024; Yuan et al., 2022). Overall, research on combination strategies has expanded rapidly since 2020, with active contributions from China, the United States, Canada, Brazil, Saudi Arabia, and Australia. These studies consistently demonstrate that non-antibacterial drugs can enhance the efficacy of traditional antibiotics against MDR *P. aeruginosa*, with the most striking effects observed for antiparasitic and anti-rheumatic agents combined with colistin (Table 2).

**TABLE 2:** Drug repurposing for synergistic antibacterial activity against drug-resistant *Pseudomonas aeruginosa*.

No.	Repurpose drug (current application)	Synergy with	PA of MIC_Rep_ (μg/ml)	PA of MIC_com_ (μg/ml)	PA of MIC_syn_ (μg/ml)	PA of MIC_com_ (μg/ml)	FICI	PA types	Antibacterial activity	Methods or models	Antibacterial effect/ mechanism	Anti-biofilm activity	Anti-QS or anti-virulence	Country of study	Year	Refs
1	Auranofin (antirheumatics)	Colistin and ceftazidime	NR	0.5	NR	2 and 8	NR	MDR	MDR-*E. coli*, MDR-*K. pneumoniae*, MDR-*A. baumannii*	*In vitro*	NR	NR	NR	USA	2016	Feng et al., 2021; Sun et al., 2016b
		Colistin and rifabutin	NR	0.5	NR	2 and 0.2	NR	MDR	MDR-*E. coli*, MDR-*K. pneumoniae*, MDR-*A. baumannii*	*In vitro*	NR	NR	NR	USA	2016	
		Colistin	512	0.5–8	64–512	2–4	0.02–0.63	XDR	MDR/XDR-*E. coli*, MDR/XDR-*K. pneumoniae*, MDR/XDR-*A. baumannii*	*In vitro*; peritoneal infection mice model (*K. pneumoniae*)	Cellular structural alterations	NR	NR	China	2021	
2	Celecoxib (non-steroidal anti-inflammatory)	Colistin	>256	16	NR	0.0625–0.25	NR	MDR	Single: MRSA, VRE, VRSA, VISA; combination: DR/MDR-*K. pneumoniae*, DR/MDR-*A. baumannii*	*In vitro*; MRSA infected *Caenorhabditis elegans* model; MRSA skin infection mice model	Dose-dependent inhibition of RNA, DNA, and protein synthesis	NR	NR	USA	2015	Thangamani et al., 2015b
3	Closantel (anthelmintic)	Colistin	256 or >256	1–4	4–1024	0.125–2	0.002–0.516	MDR	MDR-*E. coli*, MDR-*K. pneumoniae*, MDR-*A. baumannii*	*In vitro*	NR	NR	NR	Canada	2019	Ding et al., 2024; Domalaon et al., 2019c
		Colistin	>256	0.5	128	1	0.009	XDR	MDR-*E. coli*, MDR-*K. pneumoniae*	*In vitro*; cutaneous infection mice model (*P. aeruginosa*)	NR	NR	NR	China	2024	
4	Dimetridazole (antiprotozoal)	Polymyxin B	NR	50 μM	NR	1	NR	DR	NR	*In vitro*; *Caenorhabditis elegans* model (*P. aeruginosa*); pulmonary infection mice model (*P. aeruginosa*)	Influence QS	Dimetridazole reduced the formation of biofilms	Dimetridazole significantly reduced the production of protease and *P. aeruginosa*	China	2022	Yuan et al., 2022
		Kanamycin	NR	50 μM	NR	50	NR	DR	NR	*In vitro*	NR	NR	NR	China	2022	
5	Disulfiram (alcoholism)	Sulfamethoxazole/ trimethoprim	512	32	128	32	0.31	MDR	MDR-*A. baumannii*	*In vitro*	NR	NR	NR	Brazil	2022	Serafin et al., 2020
6	Doxazosin (hypertension)	Ciprofloxacin	4	1	NR	200	0.25	DR	NR	*In vitro*; abdominal cavity infection mice model (*P. aeruginosa*)	Anti-QS and anti-virulence activities	Doxazosin significantly diminished the biofilm formation	Doxazosin suppressed QS-regulated pigment and virulence in bacteria and downregulated QS genes in *P. aeruginosa*	Saudi Arabia	2023	Elfaky et al., 2023
		Cefoperazone	256	64	NR	200	0.25	DR	NR	*In vitro*	NR	NR	NR	Saudi Arabia	2023	
		Amoxicillin/ clavulinic acid	1024	512	NR	200	0.5	DR	NR	*In vitro*	NR	NR	NR	Saudi Arabia	2023	
		Imipenem	8	4	NR	200	0.5	DR	NR	*In vitro*	NR	NR	NR	Saudi Arabia	2023	
		Gentamycin	32	16	NR	200	0.5	DR	NR	*In vitro*	NR	NR	NR	Saudi Arabia	2023	
7	Fluoxetine (antipsychotics)	Meropenem	256 mcg/ml	NR	NR	NR	0.16	MDR	DR/MDR-*K. pneumoniae*, DR/MDR-*A. baumannii*	*In vitro*	NR	NR	NR	USA	2024	Ahmed et al., 2024; de Melo Guedes et al., 2024
		Fosfomycin	256–512 mcg/ml	NR	NR	NR	0.19–0.5	MDR	DR/MDR-*K. pneumoniae*, DR/MDR-*A. baumannii*	*In vitro*	NR	NR	NR	USA	2024	
		Polymyxin B	256–512 mcg/ml	NR	NR	NR	0.19–0.5	MDR	DR/MDR-*K. pneumoniae*, DR/MDR-*A. baumannii*	*In vitro*	NR	NR	NR	USA	2024	
		Ciprofloxacin	78.1–625	39–312.5	16–256	8–128	NR	MDR	NR	*In vitro*	NR	NR	NR	Brazil	2024	
		Gentamicin	78.1–625	39–312.5	2–256	1–128	NR	MDR	NR	*In vitro*	NR	NR	NR	Brazil	2024	
		Meropenem	78.1–625	39–312.5	8–256	4–128	NR	MDR	NR	*In vitro*	NR	NR	NR	Brazil	2024	
8	Mitomycin C (anticancer)	Tobramycin and ciprofloxacin	2–4	0.031–0.25	16–256	2–16	0.141–0.187	MDR	MDR-*E. coli*, MDR-*E. cloacae*, MDR-*K. pneumoniae*, MDR-*A. baumannii*	*In vitro*	NR	NR	NR	Canada	2019	Domalaon et al., 2019a; Svedholm et al., 2024
		Pentamidine	0.125–8	0.031–4	16–512	4–64	0.25–0.5	MDR	NR	*In vitro*; infected *Galleria mellonella* larvae model (*P. aeruginosa*)	Mitomycin C is a substrate of the RND efflux pumps	NR	NR	Sweden	2024	
		Gentamicin	0.125–8	1	1–128	0.25	0.5	MDR	NR	*In vitro*; infected *Galleria mellonella* larvae model (*P. aeruginosa*)	Mitomycin C is a substrate of the RND efflux pumps	NR	NR	Sweden	2024	
9	Niclosamide (anthelmintic)	Colistin	2–512 or >512	0.125–4	0.25–2	0.008–0.125	0.001–0.502	MDR XDR	MDR/XDR-*E. coli*, MDR/XDR-*K. pneumoniae*, MDR/XDR-*A. baumannii*	*In vitro*	NR	NR	NR	Canada	2019	Domalaon et al., 2019b; Lu et al., 2022
		polymyxin B	NR	2	NR	2	0.01	MDR	NR	*In vitro*	NR	NR	NR	China	2022	
10	Oxyclozanide (anthelmintic)	Colistin	32–256	0.5–32	4–1024	0.062–1	0.008–0.375	MDR XDR	MDR-*E. coli*, MDR-*K. pneumoniae*, MDR-*A. baumannii*	*In vitro*	NR	NR	NR	Canada	2019	Ayerbe-Algaba et al., 2019; Domalaon et al., 2019c
		Colistin	128–256	2	16–64 or >512	0.25–32	NR	XDR	MDR/XDR-*K. pneumoniae*, MDR/XDR-*A. baumannii*	*In vitro*	Disruption of the bacterial cell envelope (*P. aeruginosa*)	NR	NR	Spain	2019	
11	Pentamidine (antimalarial)	Mitomycin C	12 μM	1.5–6 μM	>256	64–128	0.25–0.38	MDR	MDR-*E. coli*, MDR-*E. cloacae*, MDR-*A. baumannii*, MDR-*K. pneumoniae*	*In vitro*; *Caenorhabditis elegans* model (*E. cloacae*)	NR	NR	NR	China	2020	Wu et al., 2020
		Mefloquine	>256 μM	128–256 μM	128 or >256	64–128	0.38–0.5	MDR	MDR-*E. coli*, MDR-*E. cloacae*, MDR-*A. baumannii*, MDR-*K. pneumoniae*	*In vitro*	NR	NR	NR	China	2020	
12	Phosphono- formate (antiviral)	Fosfomycin	128	32–64	NR	167 μM–5 mM	NR	DR	DR-*E. coli*, DR-*K. pneumoniae*	*In vitro*	NR	NR	NR	USA	2017	Ito et al., 2017
13	Pixantrone (aggressive B-cell non-Hodgkin’s lymphoma)	Rifampin	16–64	8–16	128	32	NR	XDR	MDR/XDR-*E. coli*, MDR/XDR-*K. pneumoniae*, MDR/XDR-*A. baumannii*	*In vitro*; peritonitis-sepsis mice model (*E. coli*)	Disrupted flagellum assembly, caused irreversible ROS buildup, and impaired proton motive force	NR	NR	China	2022	She et al., 2022
14	Promethazine (antihistamines)	Ciprofloxacin	195.3–781.2	97.5–390.5	16–256	4–128	NR	MDR	NR	*In vitro*	NR	NR	NR	Brazil	2024	de Melo Guedes et al., 2024
		Gentamicin	195.3–781.2	97.5–390.5	2–256	0.25–128	NR	MDR	NR	*In vitro*	NR	NR	NR	Brazil	2024	
		Meropenem	195.3–781.2	97.5–390.5	2–256	1–128	NR	MDR	NR	*In vitro*	NR	NR	NR	Brazil	2024	
15	Rafoxanide (anthelmintic)	Colistin	16–256 or >256	0.5–8	4–1024	0.062–0.5	0.002–0.156	MDR	MDR-*E. coli*, MDR-*K. pneumoniae*, MDR-*A. baumannii*	*In vitro*	NR	NR	NR	Canada	2019	Domalaon et al., 2019c
16	Raloxifene (selective estrogen receptor modulators)	Polymyxin B	64 or >128	NR	1–64 or >128	NR	0.14–0.52	MDR XDR	MDR/XDR-*K. pneumoniae*, MDR/XDR-*A. baumannii*	*In vitro*	Synergistically induced GFP release, membrane depolarization, permeabilization, ROS increase, and outer membrane damage (*P. aeruginosa*)	Anti-biofilm activity (*P. aeruginosa*)	NR	Australia	2017	Hussein et al., 2017
17	Ribavirin (antiviral)	Polymyxin B	NR	50 μM	NR	1.25	NR	DR	NR	*In vitro*; *Caenorhabditis elegans* model (*P. aeruginosa*); pulmonary infection mice model (*P. aeruginosa*)	Influence QS	Ribavirin reduced the formation of biofilms	Ribavirin significantly reduced the production of protease and *Pseudomonas aeruginosa*	China	2022	Yuan et al., 2022
		Meropenem	NR	50 μM	NR	20	NR	DR	NR	*In vitro*	NR	NR	NR	China	2022	
		Kanamycin	NR	100 μM	NR	60	NR	DR	NR	*In vitro*	NR	NR	NR	China	2022	
18	Rifabutin (antituberculosis)	Colistin and imipenem	NR	0.08	NR	2.43 and 4.8	NR	MDR	MDR-*E. coli*, MDR-*K. pneumoniae*, MDR-*A. baumannii*	*In vitro*	NR	NR	NR	USA	2016	Sun et al., 2016b
19	Sildenafil (idiopathic pulmonary arterial hypertension)	Polymyxin B	3.12–6.25 mg/ml	NR	NR	NR	0.063	DR	NR	*In vitro*	NR	Inhibited and eradicated biofilms, reducing total biomass (*P. aeruginosa*)	NR	Brazil	2024	Barin et al., 2024
		Cefepime	3.12–6.25 mg/ml	NR	NR	NR	0.093	DR	NR	*In vitro*	NR	Inhibited and eradicated biofilms, reducing total biomass (*P. aeruginosa*)	NR	Brazil	2024	
		Imipenem	3.12–6.25 mg/ml	NR	NR	NR	0.187	DR	NR	*In vitro*	NR	Inhibited and eradicated biofilms, reducing total biomass (*P. aeruginosa*)	NR	Brazil	2024	
20	Simvastatin (antihyper- lipidemic)	Colistin	>256	16–32	0125–0.5	0.0625–0.25	NR	DR MDR	Single: MRSA, VRE, VRSA, VISA; combination: DR/MDR-*E. coli*, DR/MDR-*K. pneumoniae*, DR/MDR-*A. baumannii*	*In vitro*; MRSA skin infection mice model	Selective interference of bacterial protein synthesis (*E. coli*)	Excellent anti-biofilm activity (*Staphylococcal*)	Inhibits bacterial toxin production (*Staphylococcal*)	USA	2015	Thangamani et al., 2015a
21	Tamoxifen (selective estrogen receptor modulators)	Polymyxin B	64 or >128	NR	1–64 or >128	NR	0.06–0.5	MDR XDR	MDR/XDR-*K. pneumoniae*, MDR/XDR-*A. baumannii*	*In vitro*	NR	NR	NR	Australia	2017	Hussein et al., 2017
22	Triclabendazole (anthelmintic)	Polymyxin B	>256	0.125–4	0.5	0.06–0.25	0.25	DR	Single: MRSA, VRE; combination: DR-*E. coli*, DR-*K. pneumoniae*, DR-*A. baumannii*	*In vitro*; sepsis mice model (*S. aureus*)	NR	NR	NR	Australia	2021	Pi et al., 2021
23	Triclosan (antibiotic)	Tobramycin	NR	100 μM	NR	50 μM	NR	DR	NR	*In vitro*	NR	Anti-biofilm activity (*P. aeruginosa*)	NR	USA	2018	Maiden et al., 2018

Com, combination; DR, drug resistance; FICI, Fractional Inhibitory Concentration Index; MDR, multidrug resistance; MIC, minimum inhibitory concentration; NR, no reported; PA, *P. aeruginosa*; QS, quorum sensing; Refs, references; Rep, repurpose; XDR, extensively drug resistant.

#### Synthesis of patterns across drug classes

Beyond individual agents, broader mechanistic themes emerged across drug classes. Disruption of bacterial membrane integrity or permeability was among the most frequently reported strategies, highlighting membrane destabilization as a central antibacterial mechanism. Efflux pump inhibition was another recurrent feature, consistently associated with improved susceptibility to conventional antibiotics. Many agents also targeted virulence regulation, particularly quorum sensing and biofilm formation, underscoring the value of attenuating pathogenicity as a complementary therapeutic approach. Finally, a substantial proportion of studies reported synergistic activity between repurposed drugs and standard antibiotics, emphasizing the translational potential of combination therapies for extending the utility of existing antimicrobials. Taken together, these findings suggest that the most promising directions for drug repurposing against ESKAPE pathogens lie in targeting bacterial membranes, efflux systems, and virulence traits, particularly when integrated with conventional antibiotics to maximize efficacy.

## Discussion

### Therapeutic challenges in drug-resistant ESKAPE infections

Globally, antibiotic resistance has become a major public health threat, with ESKAPE pathogens representing typical MDR bacteria that cause severe infections and are extremely difficult to treat (Yu et al., 2020). Their resistance mechanisms are diverse and synergistic, including the production of inactivating enzymes (e.g., extended-spectrum β-lactamases in *Klebsiella pneumoniae* hydrolyzing the β-lactam ring), alterations of antibiotic targets (e.g., penicillin-binding protein changes in *Staphylococcus aureus*) (Fonseca et al., 2012), enhanced efflux pump activity (e.g., MexAB-OprM system in *P. aeruginosa*) (Sui et al., 2012), and biofilm formation that impedes drug penetration and reduces bacterial metabolic activity (Ribeiro et al., 2016). Current therapies are limited: polymyxins remain the last-line option against MDR Gram-negative bacteria but are restricted by nephrotoxicity (Nang et al., 2021); the development of new antibiotics is slow, costly, and inefficient; phage therapy has shown variable efficacy in drug-resistant *P. aeruginosa* infection depending on bacterial, phage, and host factors (Kortright et al., 2019; Wang et al., 2006a,b); and the clinical use of antimicrobial peptides or anti-virulence compounds is constrained by stability and mechanistic challenges (Allen et al., 2014; Mahlapuu et al., 2016; Theuretzbacher and Piddock, 2019). Against this backdrop, drug repositioning offers a promising strategy to combat MDR ESKAPE pathogens, as it leverages existing pharmacological and toxicological knowledge of approved drugs, thus reducing development costs and shortening approval timelines compared with *de novo* antibiotics (Konreddy et al., 2019; Peyclit et al., 2019; Wang et al., 2006a).

### Comparison with similar reviews

Recent systematic and narrative reviews have extensively explored the potential of drug repurposing against MDR pathogens. For example, Liu et al. (2021) concluded that combining non-antibiotic drugs with existing antibiotics can enhance therapeutic efficacy and inhibit the evolution of resistance. Similarly, Boyd et al. (2021) emphasized the translational advantages of drug repurposing by reducing development costs and establishing safety, but also cautioned that clinical evidence is limited. Our findings are consistent with these observations, demonstrating that many non-antibiotic drugs (e.g., anticancer, anti-inflammatory, and antiparasitic agents) exhibit synergistic antimicrobial activity when combined with traditional antibiotics. Notably, Gardy and Loman (2018) reported that structural motifs such as aromatic rings contribute to the antimicrobial activity of non-antibiotic drugs, consistent with our findings for several compounds.

A major strength of this study lies in its combination of bibliometric analysis and systematic review, resulting in a more comprehensive assessment than either approach alone. Bibliometric analysis provides a macroscopic perspective, revealing global research trends, hotspots, and collaborative networks in the field of drug repurposing to combat antimicrobial resistance. The systematic review, on the other hand, provides a microscopic level of evidence synthesis, summarizing experimental data using strict inclusion criteria and systematically analyzing drug efficacy, mechanisms of action, and synergies with conventional antibiotics. By combining these two approaches, this study, based on bibliometric analysis, identified drug-resistant *P. aeruginosa* as a research hotspot among ESKAPE pathogens. Subsequently, a systematic review was used to include as many drug repurposing studies of drug-resistant *P. aeruginosa* as possible. This approach not only provides a more robust summary of the existing evidence but also identifies research gaps and emerging directions, providing insights for future research. This dual approach ensures the value of the research findings to a broad audience, including researchers, clinicians, and policymakers, and enhances their translational relevance.

### Research status of drug repositioning against drug-resistant ESKAPE pathogens

This study conducted a bibliometric analysis on the field of drug repositioning of drug-resistant ESKAPE pathogens, and grasped the current status, development trends, and academic value of the field through the research results of countries, institutions, authors, journals, documents, and keywords. From the perspective of literature publication and citation trends, the growth rate of annual publications has increased year by year since 2008, reaching a peak in 2024, indicating that the research activity in this field continues to rise. Although the data for 2025 is not yet complete, the trend of sustained growth has emerged. The country/region contribution shows that the United States occupies an academic leadership position in the field of drug repositioning of drug-resistant ESKAPE pathogens, followed by China and India, with the highest annual growth rate, highlighting the rise of Asian countries in this field. The dominance of the United States may be explained by its early and sustained investment in AMR research, robust federal funding, and the establishment of global research networks (Wasan et al., 2023). By contrast, China’s rapid rise reflects strong governmental policies such as the “Healthy China 2030” strategy and heavy investment in translational infectious disease research (Chen et al., 2019; Jiang and Jiang, 2021), while India’s contribution is likely driven by the high clinical burden of MDR infections, which has spurred urgent domestic research initiatives (Luz et al., 2022). In terms of international cooperation, Australia, Egypt, and South Korea show high levels of collaboration, suggesting that countries with smaller research systems strategically leverage international partnerships to amplify their impact, consistent with previous bibliometric observations that international collaboration often enhances citation performance and research visibility (Adams and Szomszor, 2024; Duarte et al., 2025; Low and Ng, 2011).

The analysis of institutional contributions shows that the United States, Australia and India contributed most prominently to drug relocalization studies against ESKAPE pathogens. This pattern may be closely related to these countries’ national policies to prioritize antibacterial resistance (AMR), strong public research funding, and the more severe threat of clinical drug resistance (World Health Organization [WHO], 2021). The close core cooperation network formed by Purdue University and Monash University highlights the core role of interdisciplinary and international cooperation in responding to global health challenges; the former’s advantages in medicinal chemistry and discovery form a strong alliance with the latter’s expertise in microbiology and clinical translation (Brown and Wright, 2016). This result strongly suggests that encouraging similar efficient cross-border and cross-institutional cooperation models in the future will be an important strategy to accelerate the discovery of new therapies against drug-resistant bacteria (Theuretzbacher et al., 2020).

Through author analysis of authoritative experts in the field and their cooperation teams, it was found that Li, Jian and Velkov, Tony ranked first in the number of publications, and Li, Jian had the highest H-index; in terms of author cooperation, the field of drug repositioning of drug-resistant ESKAPE pathogens mainly formed two major author cooperation teams, and the team of Li, Jian, Velkov, Tony made continuous contributions to the research of drug repositioning of drug-resistant ESKAPE pathogens, becoming an authoritative force in the field and having an outstanding academic status.

### Research hotspots of drug repositioning of drug-resistant ESKAPE pathogens

The field of drug repositioning for drug-resistant ESKAPE pathogens started late, but has entered a period of rapid development since 2018, and has attracted much attention from the medical science community. As the problem of drug resistance of ESKAPE pathogens has gradually become prominent, researchers have begun to make preliminary attempts at drug repositioning methods. In the early days, they focused on the basic characteristics of ESKAPE pathogens and studied biological treatments. Recently, they have mainly turned to the analysis of drug resistance mechanisms (QS and biofilm intervention have become one of the new research trends), which is helpful for drug discovery, exploring new drug targets, exploring drugs with effective antibacterial activity, and improving the targetedness of drug repositioning.

Hot spot analysis results suggest that drug resistance and *P. aeruginosa* are the most concerned topics in the field of drug repositioning for drug-resistant ESKAPE pathogens. *P. aeruginosa* is a conditionally pathogenic bacterium and a common pathogen of community-acquired and hospital-acquired infections. It poses a serious threat to patients with cystic fibrosis (CF) or immunocompromised diseases such as acquired immunodeficiency syndrome (AIDS) or cancer (De Oliveira et al., 2020; Peterson and Kaur, 2018; She et al., 2022). Data from 2019 showed that of the 4.95 million deaths caused by MDR bacteria, more than 250,000 deaths were related to *P. aeruginosa* (She et al., 2022). It is well known that 30% of pediatric infections and up to 80% of adult CF infections are caused by *P. aeruginosa*, posing a major challenge to the medical community. It has been designated as one of the highest priority pathogens by the WHO and has attracted much attention from scholars (Nang et al., 2021; Wang et al., 2006b). Therefore, this study conducted a systematic review to comprehensively identify and summarize the non-antibiotic drugs reported in the literature that are effective against drug-resistant *P. aeruginosa* and their potential mechanisms of action.

### Drug repositioning against drug-resistant *P. aeruginosa*

#### Antibacterial mechanisms

Food and Drug Administration (FDA)-approved non-antimicrobial drugs act on drug-resistant *P. aeruginosa* through a variety of mechanisms. One mechanism is to change the permeability of bacterial membranes, making it easier for antimicrobial drugs to penetrate. For example, aminoquinoline drugs can disrupt the cell membrane potential, causing the proton motive force (PMF) to collapse, thereby enhancing the activity of other antibiotics (such as rifampicin) (Li et al., 2024). Another mechanism is to inhibit efflux pumps. Brazilian scholars have shown that FDA-approved drugs such as promethazine and fluoxetine can act as efflux pump inhibitors (EPIs) to increase the accumulation of drugs in *P. aeruginosa* cells and significantly reduce the metabolic activity of biofilm (de Melo Guedes et al., 2024). In addition, some non-antimicrobial drugs can also interfere with bacterial metabolism and metabolic pathways. For example, anticancer drugs such as 5-fluorouracil, cisplatin, and mitomycin C can bind to DNA or inhibit RNA synthesis, indirectly inhibiting bacterial proliferation (Chadha et al., 2024; Yuan et al., 2022).

It is worth emphasizing that many repurposed drugs also have the function of inhibiting QS and anti-virulence factors. The anthelmintic niclosamide has been shown to potently inhibit the QS system of *P. aeruginosa*, and its mechanism includes inhibiting the synthesis of Acyl-Homoserine Lactones (AHL) signaling molecules and downregulating about 250 QS-related genes (Imperi et al., 2013). Phenotypically, niclosamide significantly reduces the production of elastase, pyocyanin, and rhamnolipids, while inhibiting bacterial motility and biofilm formation. In the insect infection model, the drug can prevent infection at a dose far lower than that of antibiotics (Imperi et al., 2013). Other drugs (such as certain kinase inhibitors or antidepressants) have also been shown to affect QS or virulence regulatory networks, but their molecular mechanisms remain to be further studied (Gad et al., 2024; Karine de Sousa et al., 2018; Neville et al., 2021).

In general, these drugs reshape the drug resistance network of *P. aeruginosa* through multiple mechanisms such as penetrating the bacterial outer membrane, inhibiting efflux pumps, disrupting metabolism or QS pathways, and destroying biofilms. Many drugs have multiple targets, such as the production of reactive oxygen species (such as mefloquine), which can not only destroy the cell membrane but also downregulate virulence factors and biofilm-related genes (Li et al., 2023). This “multi-point attack” strategy is of great significance for reversing the drug resistance of MDR/XDR strains.

#### Drug screening strategies

Researchers have used a variety of methods to screen FDA-approved non-antimicrobial drugs against *P. aeruginosa*. Among them, computer virtual screening is a common strategy. For example, Vieira et al. (2022) performed molecular docking and virtual screening on the QS regulatory protein MvfR (PqsR) to screen potential inhibitors from the FDA drug library. High molecular binding drugs were then further evaluated by molecular dynamics simulation and free energy calculation. Such methods can predict QS inhibitors at an early stage and reduce the cost of experimental screening. High-throughput *in vitro* screening is also a common method. Researchers used a library of MDR *P. aeruginosa* strains to test the growth inhibitory activity of drugs and their combined effects with antibiotics. For example, Li et al. (2023) collected multiple carbapenem-resistant *P. aeruginosa* strains and tested the antibacterial effects of mefloquine alone and in combination, and found that the combination of the two showed significant synergistic antibacterial effects. Svedholm et al. (2024) also found through checkerboard and bactericidal curve experiments that the combination of mitomycin C and pentamidine or gentamicin can produce a synergistic inhibitory effect on multiple strains of drug-resistant *P. aeruginosa.* In addition, animal infection models are further verification methods for evaluating drug efficacy and toxicity. For example, Svedholm et al. (2024) used the *Galleria mellonella* infection model to verify the combined efficacy of mitomycin C and pentamidine, and the results showed that the survival rate was significantly improved compared with a single drug. Li et al. (2023) used a mouse peritoneal infection model to confirm that mefloquine combination regimen can effectively control carbapenem-resistant infections *in vivo*. In summary, drug repurposing screening strategies usually combine computational prediction, *in vitro* screening and animal verification multi-level screening processes to increase the probability of successful clinical transformation.

#### Biofilm intervention

*Pseudomonas aeruginosa* biofilms are a major cause of chronic and refractory infections. Therefore, both the prevention of biofilm formation and the disruption of established biofilms represent key therapeutic strategies (Costerton et al., 1999). One common approach is to inhibit biofilm formation. Several non-antibiotic drugs, such as vitamin C and promethazine, have been shown to interfere with initial bacterial adhesion and inhibit polysaccharide matrix synthesis, thereby reducing biofilm formation rates (Choudhury et al., 2022; Dawan and Ahn, 2022). Another strategy focuses on promoting the disruption of mature biofilms. This can be achieved by enhancing the penetration of antibiotics or host immune effectors into the biofilm matrix. For example, Barin et al. (2024) showed that sildenafil citrate enhanced the antibacterial effects of cefepime, imipenem, and polymyxin against *P. aeruginosa*. It also effectively eradicated biofilms, with a reduction rate of up to 83.8%. Atomic force microscopy confirmed its antibiofilm activity by showing decreased biofilm thickness and surface roughness (Barin et al., 2024). Di Bonaventura et al. (2023) discovered through high-throughput screening methods that tirazamin and tavaborole can actively disperse the pre-formed biofilms of drug-resistant *P. aeruginosa*. Additionally, targeting the synthesis of biofilm matrix components or activating biofilm-degrading enzymes (e.g., depolymerases) has shown potential in promoting biofilm clearance (Ramakrishnan et al., 2022). Recent studies have also shown that combining biofilm-interfering agents with conventional antibiotics significantly reduces the viability of biofilm-embedded bacteria (Hawas et al., 2022).

### Challenges and strategies for clinical transformation

Despite rapid research progress, drug repurposing faces multiple obstacles to clinical transformation. Toxicity issues are the first to bear the brunt: non-antimicrobial drugs often show antibacterial activity only at high concentrations, which may exceed their therapeutic window. For example, anticancer drugs are highly toxic to humans, and even when used in combination with other drugs, caution is required (Gonzalez-Fierro and Dueñas-González, 2021). Pharmacokinetic limitations are also a difficulty: some drugs cannot effectively reach the site of infection after distribution in the body, or are easily metabolized and decomposed (Drusano et al., 2015a,b; Gómara and Ramón-García, 2019). Regulatory and approval barriers: Although it is an FDA-approved drug, the new use of treating infection requires additional clinical trial data support, and the approval process is complicated and costly (Breckenridge and Jacob, 2019). The above factors make the clinical application of non-antimicrobial drugs still need to be carefully evaluated. Conducting more high-quality clinical trials to verify the efficacy and safety of repositioned drugs in humans is the key to promoting their widespread use in the clinical treatment of ESKAPE pathogen infections.

To overcome these translational barriers, several strategies have been proposed. Prodrug design can reduce systemic toxicity by ensuring that drugs are activated only under infection-specific conditions (Maria et al., 2024). Targeted delivery systems and nanocarriers, such as liposomes and nanoparticles, enhance drug stability, improve penetration, and increase drug accumulation at infection sites while reducing systemic exposure. For example, nano-curcumin has been shown to inhibit *P. aeruginosa* biofilm formation and downregulate regulatory genes, thereby improving antibacterial efficacy with lower toxicity (Sharifian et al., 2020). Similarly, curcumin–silver nanoparticles displayed strong antibacterial and antibiofilm activity against *P. aeruginosa* isolates from burn patients, supporting their potential as adjunct therapies to overcome biofilm-related tolerance (Al-Oqaili et al., 2025). Combination therapies also provide significant advantages, as exemplified by GT-1 (a novel siderophore cephalosporin) combined with GT-055 (a β-lactamase inhibitor), which demonstrated potent synergistic activity against ESKAPE pathogens both *in vitro* and *in vivo* (Halasohoris et al., 2021). Collectively, these approaches highlight the potential for integrating pharmaceutical innovation, rational drug combinations, and supportive policies to accelerate the safe and effective clinical transformation of drug repurposing against MDR pathogens.

### Platforms and libraries for drug repurposing

In addition to the mechanistic and translational strategies discussed above, several platforms and compound libraries have been established to systematically support drug repurposing efforts. DrugBank provides comprehensive pharmacological, biochemical, and clinical information on approved and investigational drugs, serving as one of the most widely used resources for repositioning research (Wishart et al., 2018). The Connectivity Map (CMap) and its associated CLUE (CMap LINCS Unified Environment) platform, developed by the Broad Institute, enable comparison of drug-induced gene expression signatures with disease-associated profiles, thereby facilitating mechanism-driven repositioning (Subramanian et al., 2017). The Library of Integrated Network-Based Cellular Signatures (LINCS) program further expands this approach by offering large-scale perturbation-response datasets that link small molecules to biological pathways and phenotypes (Koleti et al., 2018).

Curated libraries have also been developed to accelerate translational applications. The Drug Repurposing Hub (Broad Institute) is a next-generation annotated library of thousands of approved and investigational drugs with systematic profiling data (Corsello et al., 2017). Similarly, the ReFRAME library (Scripps Research) represents the most comprehensive collection of nearly 12,000 compounds with clinical or preclinical safety data, which has been applied in multiple therapeutic areas (Janes et al., 2018). These resources substantially enhance the practical utility of drug repositioning by enabling researchers to efficiently identify candidate molecules, explore drug–disease associations, and design rational combination strategies, thus bridging the gap between computational discovery and clinical validation.

### Limitations

This study has several limitations that should be acknowledged in detail. First, the bibliometric analysis was restricted to the WoSCC. Although WoSCC is widely recognized for its comprehensive citation records and compatibility with bibliometric tools such as CiteSpace, VOSviewer, and Bibliometrix, this choice may have excluded relevant publications indexed in other databases such as Scopus, Dimensions, or PubMed, thereby introducing potential selection bias. The rationale for using WoSCC is that it provides a standardized, authoritative dataset with high coverage in antimicrobial resistance and drug repurposing research, ensuring methodological rigor and reproducibility of citation-based analyses. Nonetheless, we acknowledge that future studies could integrate multiple databases to achieve broader coverage and minimize database-related bias.

Second, only English-language publications were included, which may have introduced language bias and excluded potentially relevant studies published in other languages. Third, the majority of included studies were preclinical investigations (*in vitro* or *in vivo*), with a scarcity of high-quality randomized controlled clinical trials evaluating the efficacy and safety of repurposed drugs against resistant ESKAPE pathogens. This limits the translational strength of our findings.

Fourth, heterogeneity exists among the included studies in terms of experimental designs, drug concentrations, bacterial strains, and outcome measures, which reduces the comparability and generalizability of results. Finally, although several repurposed drugs demonstrated antibacterial activity or synergistic effects with conventional antibiotics, clinical translation remains constrained by toxicity at effective concentrations, pharmacokinetic limitations, and regulatory barriers. Future efforts should therefore prioritize combining multiple bibliographic databases, conducting well-designed clinical trials, and exploring innovative delivery systems to overcome these translational challenges.

## Conclusion and future prospects

This study provides a comprehensive overview of drug repurposing efforts against MDR ESKAPE pathogens by integrating bibliometric analysis and systematic review. Bibliometric analysis revealed increasing research attention since 2008, with the United States, China, and India as leading contributors, and identified quorum sensing inhibition and biofilm disruption as emerging hotspots. The systematic review further highlighted that several non-antimicrobial drugs, including niclosamide, mitomycin C, and amlodipine, exhibit antibacterial effects through mechanisms such as disruption of membrane permeability, inhibition of efflux pumps, and attenuation of virulence factors. Many of these agents demonstrated synergistic activity when combined with conventional antibiotics, emphasizing their therapeutic promise, particularly against drug-resistant *P. aeruginosa*.

Despite these promising results, clinical translation of repurposed drugs remains constrained by toxicity at effective concentrations, pharmacokinetic barriers, and complex regulatory requirements. To address these challenges, strategies such as prodrug design, nanocarrier-based targeted delivery, and rational combination therapies offer practical avenues to enhance efficacy, reduce systemic toxicity, and accelerate the clinical application of repurposed drugs.

In conclusion, drug repurposing offers a cost-effective and time-efficient strategy to tackle the global challenge of antimicrobial resistance. By highlighting promising candidates, clarifying their mechanisms of action, and outlining strategies to overcome translational barriers, this study provides actionable insights for researchers, clinicians, and policymakers. Future efforts should prioritize innovative delivery systems, rational combination regimens, and high-quality clinical trials to accelerate the transition of repurposed drugs from bench to bedside.

## Data Availability

The original contributions presented in this study are included in this article/Supplementary material, further inquiries can be directed to the corresponding authors.
