# Correction: A bibliometric analysis and systematic review of drug repurposing against drug-resistant ESKAPE pathogens: a particular focus on *Pseudomonas aeruginosa*

**DOI:** 10.3389/fmicb.2025.1730944

**Published:** 2025-11-14

**Authors:** Frontiers Production Office

**Affiliations:** Frontiers Media SA, Lausanne, Switzerland

**Keywords:** drug repurposing, drug-resistant ESKAPE, drug-resistant *Pseudomonas aeruginosa*, bibliometric analysis, systematic review

Incorrect Funding and Acknowledgement statements were published.

The correct statements are below:

