# Supplementary material for: A bibliometric analysis and systematic review of drug repurposing against drug-resistant ESKAPE pathogens: a particular focus on Pseudomonas aeruginosa
Source: Front Microbiol. 2025 Oct 16;16:1669585. doi: 10.3389/fmicb.2025.1669585 (PMC12571837; doi:10.3389/fmicb.2025.1669585)
Supplement: Supplementary file 1 [file Table_1.docx]

**A bibliometric** **analysis and systematic review of drug repurposing against drug-resistant** **ESKAPE pathogens: A particular focus on *Pseudomonas aeruginosa***

**Supplementary materials**

**Supplementary Table 1**. Search strategy for the included databases based.

**Supplementary Table 2.** Summary of literature related to drug repurposing against drug-resistant ESKAPE pathogens

**Supplementary Table 3.** Table 1 Top 10 countries in terms of publications.

**Supplementary Table 4.** The top ten most active institutions in the field of drug-resistant ESKAPE drug relocalization.

**Supplementary Table 5.** The top ten most active authors in the field of drug-resistant ESKAPE drug relocalization.

**Supplementary Table 6.** The top 10 journals in terms of number of total publications.

**Supplementary Table 7.** The top 10 highly cited references.

**Supplemental Table 1.** Search strategy for the included databases based

| **Web of science (April 7, 2025)** | | |
| --- | --- | --- |
| **#1** | ((((((((((TS=(Enterobacteriaceae)) OR TS=(Coliform Bacilli)) OR TS=(Enterobacteria)) OR TS=(Paracolobactrum)) OR TS=(Ewingella)) OR TS=(Leclercia)) OR TS=(Sodalis)) OR TS=(Enterobacterales)) OR TS=(Enterobacter)) OR TS=(Enterobacter)) | 682,917 |
| **#2** | ((TS=(Acinetobacter baumannii)) OR TS=(Bacterium anitratum)) | 25,247 |
| **#3** | (((((((TS=(Pseudomonas aeruginosa)) OR TS=(Bacillus aeruginosus)) OR TS=(Bacillus pyocyaneus)) OR TS=(Pseudomonas pyocyanea)) OR TS=(Bacterium aeruginosum)) OR TS=(Bacterium pyocyaneum)) OR TS=(Pseudomonas polycolor)) | 165,169 |
| **#4** | ((((((TS=(Klebsiella pneumoniae)) OR TS=(Klebsiella pneumoniae aerogenes)) OR TS=(Hyalococcus pneumoniae)) OR TS=(Bacillus pneumoniae)) OR TS=(Bacterium pneumoniae)) OR TS=(Klebsiella rhinoscleromatis)) | 126,547 |
| **#5** | (TS=(Staphylococcus aureus)) | 289,403 |
| **#6** | ((TS=(Enterococcus faecium)) OR TS=(Streptococcus faecium)) | 19,412 |
| **#7** | (((TS=(Drug Repositioning)) OR TS=(Drug Repurposing)) OR TS=(Drug Rescue)) | 91,227 |
| **#8** | (TS=(Drug Resistance)) | 1,093,949 |
| **#9** | (#1 OR #2 OR #3 OR #4 OR #5 OR #6) AND #7 AND #8 | 1,395 |

| **PubMed (April 7, 2025)** | | |
| --- | --- | --- |
| **#1** | (((((((((("Enterobacteriaceae"[Mesh]) OR (Enterobacteriaceae[Title/Abstract])) OR (Coliform Bacilli[Title/Abstract])) OR (Enterobacteria[Title/Abstract])) OR (Paracolobactrum[Title/Abstract])) OR (Ewingella[Title/Abstract])) OR (Leclercia[Title/Abstract])) OR (Sodalis[Title/Abstract])) OR (Enterobacterales[Title/Abstract])) OR ("Enterobacter"[Mesh])) OR (Enterobacter[Title/Abstract]) | 475,344 |
| **#2** | (("Acinetobacter baumannii"[Mesh]) OR (Acinetobacter baumanii[Title/Abstract])) OR (Bacterium anitratum[Title/Abstract]) | 8,669 |
| **#3** | ((((((("Pseudomonas aeruginosa"[Mesh]) OR (Pseudomonas aeruginosa[Title/Abstract])) OR (Bacillus aeruginosus[Title/Abstract])) OR (Bacillus pyocyaneus[Title/Abstract])) OR (Pseudomonas pyocyanea[Title/Abstract])) OR (Bacterium aeruginosum[Title/Abstract])) OR (Bacterium pyocyaneum[Title/Abstract])) OR (Pseudomonas polycolor[Title/Abstract]) | 86,358 |
| **#4** | (((((("Klebsiella pneumoniae"[Mesh]) OR (Klebsiella pneumoniae[Title/Abstract])) OR (Klebsiella pneumoniae aerogenes[Title/Abstract])) OR (Hyalococcus pneumoniae[Title/Abstract])) OR (Bacillus pneumoniae[Title/Abstract])) OR (Bacterium pneumoniae[Title/Abstract])) OR (Klebsiella rhinoscleromatis[Title/Abstract]) | 35,985 |
| **#5** | ("Staphylococcus aureus"[Mesh]) OR (Staphylococcus aureus[Title/Abstract]) | 161,556 |
| **#6** | (("Enterococcus faecium"[Mesh]) OR (Enterococcus faecium[Title/Abstract])) OR (Streptococcus faecium[Title/Abstract]) | 9,207 |
| **#7** | ((("Drug Repositioning"[Mesh]) OR (Drug Repositioning[Title/Abstract])) OR (Drug Repurposing[Title/Abstract])) OR (Drug Rescue[Title/Abstract]) | 11,581 |
| **#8** | ("Drug Resistance"[Mesh]) OR (Drug Resistance[Title/Abstract]) | 452,215 |
| **#9** | (#1 OR #2 OR #3 OR #4 OR #5 OR #6) AND #7 AND #8 | 88 |

| **Embase (April 7, 2025)** | | |
| --- | --- | --- |
| **#1** | 'enterobacteriaceae'/exp | 714,609 |
| **#2** | 'enterobacteriaceae':ab,kw,ti OR 'coliform bacilli':ab,kw,ti OR 'enterobacteria':ab,kw,ti OR 'paracolobactrum':ab,kw,ti OR 'ewingella':ab,kw,ti OR 'leclercia':ab,kw,ti OR 'sodalis':ab,kw,ti OR 'enterobacterales':ab,kw,ti OR 'enterobacter':ab,kw,ti | 55,420 |
| **#3** | 'acinetobacter baumannii'/exp | 27,946 |
| **#4** | 'Acinetobacter baumannii':ab,kw,ti OR 'Bacterium anitratum':ab,kw,ti | 18,226 |
| **#5** | 'Pseudomonas aeruginosa'/exp | 141,151 |
| **#6** | 'Pseudomonas aeruginosa':ab,kw,ti OR 'Bacillus aeruginosus':ab,kw,ti OR 'Bacillus pyocyaneus':ab,kw,ti OR 'Pseudomonas pyocyanea':ab,kw,ti OR 'Bacterium aeruginosum':ab,kw,ti OR 'Bacterium pyocyaneum':ab,kw,ti OR 'Pseudomonas polycolor':ab,kw,ti | 98,049 |
| **#7** | 'Klebsiella pneumoniae'/exp | 69,245 |
| **#8** | 'Klebsiella pneumoniae':ab,kw,ti OR 'Klebsiella pneumoniae aerogenes':ab,kw,ti OR 'Hyalococcus pneumoniae':ab,kw,ti OR 'Bacillus pneumoniae':ab,kw,ti OR 'Bacterium pneumoniae':ab,kw,ti OR 'Klebsiella rhinoscleromatis':ab,kw,ti | 409,91 |
| **#9** | 'Staphylococcus aureus'/exp | 241,173 |
| **#10** | 'Staphylococcus aureus':ab,kw,ti | 170,353 |
| **#11** | 'Enterococcus faecium'/exp | 14,508 |
| **#12** | 'Enterococcus faecium':ab,kw,ti OR 'Streptococcus faecium':ab,kw,ti | 9,763 |
| **#13** | 'Drug Repositioning'/exp | 13,235 |
| **#14** | 'Drug Repositioning':ab,kw,ti OR 'Drug Rescue':ab,kw,ti OR 'Drug Repurposing':ab,kw,ti | 10,757 |
| **#15** | 'Drug Resistance'/exp | 427,675 |
| **#16** | 'Drug Resistance':ab,kw,ti | 131,614 |
| **#17** | (#1 OR #2 OR #3 OR #4 OR #5 OR #6 OR #7 OR #8 OR #9 OR #10 OR #11 OR #12) AND (#13 OR #14) AND (#15 OR #16) | 259 |

| **Cochrane (April 7, 2025)** | | |
| --- | --- | --- |
| **#1** | MeSH descriptor: [Enterobacteriaceae] explode all trees | 1,778 |
| **#2** | ("enterobacteriaceae" OR "coliform bacilli" OR "enterobacteria" OR "paracolobactrum" OR "ewingella" OR "leclercia" OR "sodalis" OR "enterobacterales" OR "enterobacter"):ab,kw,ti | 1,681 |
| **#3** | MeSH descriptor: [Acinetobacter baumannii] explode all trees | 40 |
| **#4** | ("Acinetobacter baumannii" OR "Bacterium anitratum"):ab,kw,ti | 18,226 |
| **#5** | MeSH descriptor: [Pseudomonas aeruginosa] explode all trees | 518 |
| **#6** | ("Pseudomonas aeruginosa" OR "Bacillus aeruginosus" OR "Bacillus pyocyaneus" OR "Pseudomonas pyocyanea" OR "Bacterium aeruginosum" OR "Bacterium pyocyaneum" OR "Pseudomonas polycolor"):ab,kw,ti | 2,391 |
| **#7** | MeSH descriptor: [Klebsiella pneumoniae] explode all trees | 142 |
| **#8** | ("Klebsiella pneumoniae" OR "Klebsiella pneumoniae aerogenes" OR "Hyalococcus pneumoniae" OR "Bacillus pneumoniae" OR "Bacterium pneumoniae" OR "Klebsiella rhinoscleromatis"):ab,kw,ti | 710 |
| **#9** | MeSH descriptor: [Staphylococcus aureus] explode all trees | 1,153 |
| **#10** | ("Staphylococcus aureus"):ab,kw,ti | 4,072 |
| **#11** | MeSH descriptor: [Enterococcus faecium] explode all trees | 50 |
| **#12** | ("Enterococcus faecium" OR "Streptococcus faecium"):ab,kw,ti | 226 |
| **#13** | MeSH descriptor: [Drug Repositioning] explode all trees | 64 |
| **#14** | ("Drug Repurposing" OR "Drug Repositioning" OR "Drug Rescue" OR "Rescue, Drug" OR "Drug Rescue" OR "Repurposing, Drug" OR "Drug Repurposing" OR "Repositioning, Drug"):ab,kw,ti | 458 |
| **#15** | MeSH descriptor: [Drug Resistance] explode all trees | 12,902 |
| **#16** | ("Drug Resistance"):ab,kw,ti | 10,501 |
| **#17** | (#1 OR #2 OR #3 OR #4 OR #5 OR #6 OR #7 OR #8 OR #9 OR #10 OR #11 OR 12) AND (#13 OR #14) AND (#15 OR #16) | 3 |

**Supplementary Table 2.** Summary of literature related to drug repurposing against drug-resistant ESKAPE pathogens

| Repurpose Drug | Repurpose Drug antibacterial | Synergistic antibacterial | *P. aeruginosa* | *K. pneumoniae* | *Enterobacteriaceae* | *A. baumannii* | *E. faecium* | *S. aureus* | Refs |
| --- | --- | --- | --- | --- | --- | --- | --- | --- | --- |
| 5-Fluorouracil | 5-Fluorouracil |  | 1 |  |  |  |  |  | [1] |
| 9-Aminoacridine | 9-Aminoacridine | Rifampin | 1 | 1 | 1 | 1 |  | 1 | [2] |
| Adapalene |  | Meropenem |  | 1 | 1 |  |  |  | [3] |
| Ambroxol | Ambroxol |  |  |  |  |  |  | 1 | [4] |
| Amitriptyline | Amitriptyline | Antibacterial drug |  | 1 |  |  |  |  | [5] |
| Amlodipine | Amlodipine |  | 1 |  |  |  |  |  | [6] |
|  | Amlodipine |  |  |  |  |  |  | 1 | [7] |
| Antidepressant |  | Ciprofloxacin |  | 1 | 1 | 1 | 1 | 1 | [8] |
| Astemizole | Astemizole |  |  |  |  |  |  | 1 | [9] |
| Atorvastatin | Atorvastatin | Tobramycin and Ciprofloxacin |  |  |  | 1 |  |  | [10] |
| Auranofin | Auranofin |  |  |  |  |  | 1 | 1 | [11] |
|  | Auranofin |  |  |  |  |  | 1 | 1 | [12] |
|  |  | Antibacterial drug |  |  |  |  | 1 | 1 | [13] |
|  | Auranofin |  |  |  |  |  |  | 1 | [14] |
|  | Auranofin |  |  |  |  |  |  | 1 | [15] |
|  | Auranofin | Colistin | 1 | 1 | 1 | 1 |  |  | [16] |
|  | Auranofin |  |  |  |  | 1 |  |  | [17] |
|  |  | Colistin |  |  | 1 |  |  |  | [18] |
|  |  | Phenethyl isothiocyanate |  |  |  |  |  | 1 | [19] |
|  |  | Pentamidine |  | 1 | 1 | 1 |  |  | [20] |
|  |  | Doripenem |  |  |  | 1 |  |  | [21] |
| Benzbromarone | Benzbromarone |  |  |  |  |  | 1 | 1 | [22] |
| Benzodiazepine |  | Ciprofloxacin |  | 1 |  | 1 | 1 |  | [23] |
| Benzydamine |  | Tetracycline |  | 1 | 1 | 1 | 1 | 1 | [24] |
| Bithionol | Bithionol |  |  |  |  |  | 1 |  | [25] |
| BMS-833923 | BMS-833923 | Colistin |  |  |  |  |  |  | [26] |
| Bumetanide | Bumetanide |  |  |  |  |  |  | 1 | [27] |
| Candesartan Cilexetil | Candesartan Cilexetil |  |  |  |  |  | 1 |  | [28] |
|  | Candesartan Cilexetil |  |  |  |  |  |  | 1 | [29] |
| Caspofungin |  | Rifampin |  |  | 1 |  |  |  | [30] |
| Celecoxib |  | Colistin | 1 | 1 | 1 | 1 |  |  | [31] |
| Ceritinib | Ceritinib |  |  |  |  |  |  | 1 | [32] |
| Cetylpyridinium chloride | | Colistin |  | 1 | 1 |  |  |  | [33] |
| Ciclopirox | Ciclopirox |  | 1 | 1 | 1 | 1 |  |  | [34] |
|  | Ciclopirox |  | 1 |  |  |  |  |  | [35] |
|  |  | Zidovudine |  | 1 | 1 | 1 |  |  | [36] |
| Cinacalcet | Cinacalcet |  |  |  |  |  |  | 1 | [37] |
| Cisplatin | Cisplatin |  | 1 |  |  |  |  |  | [1] |
| Closantel |  | Polymyxin B |  |  |  | 1 |  |  | [38] |
|  |  | Colistin | 1 | 1 | 1 |  |  |  | [39] |
| Crizotinib | Crizotinib | Clindamycin and gentamicin |  |  |  |  | 1 | 1 | [40] |
| Desloratadine | Desloratadine |  |  |  |  | 1 |  |  | [41] |
| Diclofenac | Diclofenac |  |  |  |  |  |  | 1 | [42] |
| Dimetridazole | Dimetridazole | Antibacterial drug | 1 |  |  |  |  |  | [43] |
| Diminazene aceturate |  | Streptomycin and chloramphenicol | | 1 | 1 |  |  | 1 | [44] |
| Disulfiram | Disulfiram |  |  | 1 |  | 1 | 1 | 1 | [45] |
|  |  | Vancomycin |  |  |  |  | 1 |  | [46] |
| Domiphen bromide |  | Colistin |  | 1 | 1 |  |  |  | [33] |
| Doxazosin | Doxazosin |  | 1 |  |  |  |  |  | [47] |
| Doxifluridine | Doxifluridine |  |  |  |  |  |  | 1 | [48] |
| Ebselen | Ebselen | Antibacterial drug |  |  |  |  | 1 | 1 | [49] |
|  | Ebselen |  |  |  |  |  | 1 | 1 | [50] |
|  | Ebselen |  |  |  |  |  | 1 |  | [51] |
| Eltrombopag | Eltrombopag |  |  |  |  |  |  | 1 | [52] |
| Ethinylestradiol | Ethinylestradiol |  |  |  | 1 |  |  | 1 | [53] |
| Ethyl bromopyruvate | Ethyl bromopyruvate |  |  | 1 |  | 1 | 1 | 1 | [54] |
| Etoposide | Etoposide |  | 1 |  |  |  |  |  | [1] |
| Etrasimod | Etrasimod |  |  |  |  |  | 1 | 1 | [54] |
| Fasciolicide Triclabendazole | Fasciolicide Triclabendazole | Antibacterial drug | 1 | 1 | 1 | 1 | 1 | 1 | [55] |
| Fluoxetine |  | Antibacterial drug | 1 | 1 |  | 1 | 1 | 1 | [56] |
| Four repurpose drugs | Four repurpose drugs |  |  |  |  | 1 |  |  | [57] |
| FtsZ inhibitor |  | Oxacillin |  |  |  |  |  | 1 | [58] |
| Fty720 |  |  |  | 1 | 1 | 1 |  | 1 | [59] |
| Gemcitabine | Gemcitabine | Gentamicin |  |  |  |  |  | 1 | [60] |
| Guanabenz acetate | Guanabenz acetate |  |  |  | 1 |  |  |  | [61] |
| Hexestrol | Hexestrol |  |  |  |  |  | 1 | 1 | [62] |
| Hydralazine | Hydralazine |  |  |  |  |  |  | 1 | [63] |
| Ibuprofenate | Ibuprofenate |  |  |  |  |  |  |  | [64] |
| Imipramin | Imipramin |  |  |  |  |  |  | 1 | [7] |
| Lvacaftor | Lvacaftor |  |  |  |  |  | 1 | 1 | [65] |
| Menadione | Menadione | Oxacillin |  |  |  |  |  | 1 | [66] |
| Methotrexate | Methotrexate |  |  |  | 1 |  |  |  | [67] |
| Mitomycin C | Mitomycin C |  |  |  |  | 1 |  |  | [68] |
|  | Mitomycin C | Tobramycin-Ciprofloxacin | 1 | 1 | 1 | 1 |  |  | [1] |
|  |  | Pentamidine or Gentamicin | 1 |  |  |  |  |  | [69] |
| Multiple antifungal Drugs^a^ | | Colistin |  |  | 1 |  |  |  | [70] |
| Multiple antifungal Drugs^a^ | Multiple antifungal Drugs |  |  |  |  |  |  | 1 | [71] |
| Multiple antifungal Drugs^a^ | Multiple repurpose drugs |  |  |  |  |  |  | 1 | [72] |
| Multiple antifungal Drugs^a^ | | Two antibacterial drugs | 1 | 1 | 1 | 1 |  |  | [73] |
| Multiple antifungal Drugs^a^ | Multiple repurpose drugs |  |  | 1 | 1 | 1 | 1 | 1 | [74] |
| Multiple antifungal Drugs^a^ | Multiple repurpose drugs |  |  |  |  |  | 1 | 1 | [75] |
| Multiple antifungal Drugs^a^ | Multiple repurpose drugs |  |  |  |  |  |  | 1 | [76] |
| Multiple antifungal Drugs^a^ | | Colistin | 1 | 1 | 1 | 1 |  |  | [77] |
| Multiple antifungal Drugs^a^ | Multiple repurpose drugs |  |  |  |  | 1 |  |  | [78] |
| Multiple antifungal Drugs^a^ | Multiple repurpose drugs |  |  |  |  | 1 |  |  | [79] |
| Multiple antifungal Drugs^a^ | Multiple repurpose drugs |  |  |  |  |  |  | 1 | [80] |
| Multiple antifungal Drugs^a^ | Multiple repurpose drugs |  |  |  |  |  | 1 |  | [81] |
| Multiple antifungal Drugs^a^ | Multiple repurpose drugs |  | 1 | 1 |  | 1 |  | 1 | [82] |
| Multiple antifungal Drugs^a^ | | Antibacterial drug |  |  |  | 1 |  |  | [83] |
| Multiple antifungal Drugs^a^ | |  |  |  |  |  |  | 1 | [84] |
| Multiple antifungal Drugs^a^ | | Polymyxin B |  | 1 |  | 1 |  |  | [85] |
| Multiple antifungal Drugs^a^ | Multiple repurpose drugs |  | 1 |  |  |  |  |  | [86] |
| Multiple antifungal Drugs^a^ | Multiple repurpose drugs |  | 1 |  |  |  |  |  | [87] |
| Multiple antifungal Drugs^a^ | Multiple repurpose drugs |  |  |  |  |  |  | 1 | [88] |
| Multiple antifungal Drugs^a^ | | Cefazolin |  |  |  |  |  | 1 | [89] |
| Multiple antifungal Drugs^a^ | Multiple repurpose drugs |  |  |  |  |  |  | 1 | [90] |
| Niclosamide |  | Colistin | 1 | 1 | 1 | 1 |  |  | [91] |
|  | Niclosamide |  |  |  |  |  | 1 | 1 | [92] |
|  | Niclosamide |  |  |  |  |  | 1 |  | [93] |
|  |  | Phenylarginine-β-naphthylamide | |  | 1 |  |  |  | [94] |
|  |  | Polymyxin B | 1 |  |  |  |  |  | [95] |
|  | Niclosamide |  |  |  |  |  | 1 | 1 | [96] |
|  |  | Linezolid |  |  |  |  |  | 1 | [97] |
|  |  | Colistin |  |  | 1 | 1 |  |  | [98] |
|  |  | Polymyxin B |  | 1 | 1 |  |  |  | [99] |
| NSAID | NSAID |  |  |  |  |  |  | 1 | [100] |
| Offluoxetine | Offluoxetine | Ciprofloxacin |  | 1 |  | 1 |  |  | [101] |
| Otilonium Bromide | Otilonium Bromide |  |  |  |  |  |  | 1 | [102] |
| Oxiconazole |  | Gentamicin |  |  |  |  | 1 | 1 | [103] |
| Oxyclozanide |  | Colistin | 1 | 1 |  | 1 |  |  | [104] |
| Paroxetine | Paroxetine | Ciprofloxacin |  | 1 |  | 1 |  |  | [101] |
|  | Paroxetine |  |  |  |  |  |  | 1 | [105] |
| Penfluridol | Penfluridol |  |  |  |  |  | 1 | 1 | [106] |
| Pentamidine |  | Mitomycin C or mefloquine | 1 | 1 | 1 | 1 | 1 | 1 | [107] |
| PFK-158 |  | Colistin |  | 1 | 1 |  |  |  | [108] |
| Phosphonoformate |  | Fosfomycin | 1 | 1 | 1 |  |  |  | [109] |
| Pimozide | Pimozide |  |  |  |  |  |  | 1 | [110] |
| Promethazine | Promethazine |  | 1 |  | 1 |  | 1 | 1 | [111] |
| Quinestrol | Quinestrol |  |  |  | 1 |  |  | 1 | [53] |
| Rafoxanide | Rafoxanide |  |  | 1 | 1 |  |  |  | [112] |
| Rifabutin | Rifabutin |  |  |  |  |  |  | 1 | [113] |
| Rifampicin |  | Cationic Polyurethanes |  |  | 1 |  |  |  | [114] |
| Rifampin |  | Pixantrone | 1 | 1 | 1 | 1 |  |  | [115] |
| Rifamycin |  | Polymyxin B |  |  |  | 1 |  |  | [116] |
| Risedronate | Risedronate |  |  |  | 1 |  |  |  | [67] |
| Salifungin | Salifungin |  |  |  |  |  |  | 1 | [117] |
| Selamectin | Selamectin |  |  |  |  |  |  | 1 | [118] |
| Selective estrogen receptor modulators (SERMs) | | Polymyxin B | 1 | 1 |  | 1 |  |  | [119] |
| Sertraline |  | Disulfiram | 1 |  |  | 1 |  | 1 | [120] |
| Sertraline | Sertraline |  | 1 | 1 | 1 | 1 | 1 | 1 | [121] |
| Sildenafil | Sildenafil |  | 1 |  |  |  |  |  | [122] |
| Simeprevir | Simeprevir |  |  |  |  |  |  | 1 | [123] |
|  |  | Polymyxins |  |  |  |  | 1 | 1 | [124] |
| Simvastatin | Simvastatin |  |  |  |  |  |  | 1 | [125] |
|  |  | Colistin | 1 | 1 | 1 | 1 |  |  | [126] |
|  | Simvastatin |  |  | 1 |  |  |  |  | [127] |
|  | Simvastatin |  |  |  |  |  |  | 1 | [128] |
| Tafenoquine | Tafenoquine |  |  |  |  |  |  | 1 | [129] |
| Tamoxifen |  | Antibacterial drug |  |  | 1 | 1 |  |  | [130] |
| Tavaborole | Tavaborole | Aminoglycosides |  | 1 | 1 |  |  | 1 | [131] |
| Triclosan |  | Aminoglycoside | 1 |  |  |  |  |  | [132] |
| Two repurpose drugs^b^ | Two repurpose drugs |  |  |  |  |  | 1 | 1 | [133] |
| Two repurpose drugs^b^ | Two repurpose drugs |  |  |  |  |  | 1 | 1 | [134] |
| Visomitin | Visomitin |  |  |  |  |  | 1 | 1 | [135] |
| Zidovudine | Zidovudine | Tigecycline |  | 1 | 1 |  |  |  | [136] |
|  |  | Colistin |  | 1 |  |  |  |  | [137] |
|  |  | Colistin |  | 1 | 1 |  |  |  | [138] |
|  |  | Meropenem |  | 1 |  |  |  |  | [139] |
|  |  | Antibacterial drug |  | 1 |  |  |  |  | [140] |
|  |  | Fosfomycin |  | 1 | 1 |  |  |  | [141] |
|  |  | Nitrofurantoin or omadacycline | | 1 |  |  |  |  | [43] |

**Note:** ^a^:The number of effective relocalization drugs reported in the literature is greater than two; ^b^: There are two kinds of effective relocalization drugs reported in the literature; “1”: represents being "active" against this bacterium; Refs: Reference.

| **Rank** | **Country**  **(N = 62)** | **Publications** | **Total citations** | **SCP** | **MCP** | **MCP %** | **Total link strength** |
| --- | --- | --- | --- | --- | --- | --- | --- |
| 1 | USA | 110 | 5740 | 61 | 14 | 18.7 | 69 |
| 2 | China | 84 | 1959 | 61 | 11 | 15.3 | 30 |
| 3 | India | 65 | 943 | 50 | 9 | 15.3 | 22 |
| 4 | Brazil | 27 | 406 | 22 | 2 | 8.3 | 9 |
| 5 | Italy | 25 | 581 | 18 | 4 | 18.2 | 11 |
| 6 | Spain | 24 | 563 | 14 | 6 | 30 | 11 |
| 7 | Australia | 22 | 788 | 6 | 13 | 68.4 | 24 |
| 8 | Canada | 21 | 1093 | 12 | 4 | 25 | 12 |
| 9 | Egypt | 21 | 433 | 7 | 10 | 58.8 | 24 |
| 10 | Saudi Arabia | 20 | 218 | 6 | 2 | 25 | 23 |

**Supplementary Table 3.** Top 10 countries in terms of publications.

| **Rank** | **Institution (N = 807)** | **Publications** | **Total citations** | **Total link strength** | **Country** |
| --- | --- | --- | --- | --- | --- |
| 1 | Purdue University | 12 | 797 | 13 | USA |
| 2 | Monash University | 12 | 556 | 29 | Australia |
| 3 | King Abdulaziz University | 9 | 149 | 21 | Saudi Arabia |
| 4 | Zagazig University | 9 | 186 | 30 | Egypt |
| 5 | Academy of Scientific and Innovative Research | 8 | 67 | 6 | India |
| 6 | Univ Melbourne | 8 | 401 | 20 | Australia |
| 7 | Panjab University | 7 | 105 | 1 | India |
| 8 | Harvard University | 6 | 1949 | 11 | USA |
| 9 | University Seville | 6 | 114 | 11 | Spain |
| 10 | Anhui Med University | 5 | 16 | 5 | China |

**Supplementary Table 4.** The top ten most active institutions in the field of drug-resistant ESKAPE drug relocalization.

**Supplementary Table 5.** The top ten most active authors in the field of drug-resistant ESKAPE drug relocalization.

| **Rank** | **Author (N = 2570)** | **Publications** | **Total citations** | **Total link strength** | **H-index** |
| --- | --- | --- | --- | --- | --- |
| 1 | Li, Jian | 8 | 388 | 29 | 8 |
| 2 | Velkov, Tony | 8 | 383 | 30 | 7 |
| 3 | Page, Stephen | 4 | 77 | 24 | 4 |
| 4 | Trott, Darren | 4 | 77 | 24 | 4 |
| 5 | Garg, Sanjay | 3 | 72 | 20 | 3 |
| 6 | Khazandi, Manouchehr | 3 | 72 | 20 | 3 |
| 7 | Ogunniyi, Abiodun | 3 | 57 | 18 | 4 |
| 8 | Pi, Hongfei | 3 | 59 | 19 | 3 |
| 9 | Venter, Henrietta | 3 | 59 | 19 | 3 |
| 10 | Bergen, Phillip | 2 | 56 | 11 | 2 |

**Supplementary Table 6.** The top 10 journals in terms of number of total publications.

| **Rank** | **Journal (N = 182)** | **IF/JCR quartile (2023-2024)** | **Publications** | **Total citations** |
| --- | --- | --- | --- | --- |
| 1 | Frontiers in Microbiology | 4.0/Q2 | 30 | 829 |
| 2 | Antibiotics-Basel | 4.3/Q1 | 25 | 401 |
| 3 | Antimicrobial Agents and Chemotherapy | 4.1/Q1 | 18 | 673 |
| 4 | Microbiology Spectrum | 3.7/Q2 | 13 | 74 |
| 5 | International Journal of Antimicrobial Agents | 4.9/Q1 | 11 | 503 |
| 6 | Journal of Antimicrobial Chemotherapy | 3.9/Q1 | 10 | 356 |
| 7 | Plos One | 2.9/Q1 | 10 | 392 |
| 8 | Microbial Pathogenesis | 3.3/Q1 | 9 | 107 |
| 9 | Frontiers in Cellular and Infection Microbiology | 4.6/Q1 | 8 | 188 |
| 10 | ACS Infectious Diseases | 4.1/Q1 | 7 | 63 |

**Supplementary Table 7** The top 10 highly cited references

| **Rank** | **Title** | **Year, Journal** | **First author** | **Total Citation** | **TC per Year** |
| --- | --- | --- | --- | --- | --- |
| 1 | [Antibiotics for Emerging Pathogens](https://webofscience.clarivate.cn/wos/woscc/full-record/WOS:000269382300030) | 2009, Science | Fischbach, MA | [1,421](https://webofscience.clarivate.cn/wos/woscc/citing-summary/268596099?type=refid) | 83.59 |
| 2 | Rescuing the Last-Line Polymyxins: Achievements and Challenges | 2021, Pharmacological Reviews | Nang, MC | 251 | 50.2 |
| 3 | Opportunities for natural products in 21st century antibiotic discovery | 2017, Natural Product Reports | [Wright, GD](https://webofscience.clarivate.cn/wos/author/record/2401665) | 243 | 27 |
| 4 | Identification of novel antimicrobials using a live-animal infection model | 2006, Proceedings of the National Academy of Sciences of the United States of America | Moy, Terence | 240 | 12 |
| 5 | [Antibiotic Adjuvants: Rescuing Antibiotics from Resistance](https://webofscience.clarivate.cn/wos/woscc/full-record/WOS:000386644800004) | 2016, Trends in Microbiology | [Wright, GD](https://webofscience.clarivate.cn/wos/author/record/2401665) | 233 | 23.3 |
| 6 | Synergistic interactions of phytochemicals with antimicrobial agents: Potential strategy to counteract drug resistance | 2019, Chemico-Biological Interactions | Ayaz, M | 199 | 28.43 |
| 7 | Drug repurposing as an alternative for the treatment of recalcitrant bacterial infections | 2015, Frontiers in Microbiology | Rangel-Vega, A | 137 | 12.45 |
| 8 | Defeating Antibiotic-Resistant Bacteria: Exploring Alternative Therapies for a Post-Antibiotic Era | 2020, International Journal of Molecular Sciences | Wang, CH | 133 | 22.17 |
| 9 | Repurposing Salicylanilide Anthelmintic Drugs to Combat Drug Resistant Staphylococcus aureus | 2015, Plos one | Rajamuthiah, R | 177 | 10.64 |
| 10 | Repurposing the antimycotic drug flucytosine for suppression of Pseudomonas aeruginosa pathogenicity | 2013, Proceedings of the National Academy of Sciences of the United States of America | Imperi, F | 116 | 8.92 |

**Reference**

1. Domalaon, R., et al., *Repurposed Antimicrobial Combination Therapy: Tobramycin-Ciprofloxacin Hybrid Augments Activity of the Anticancer Drug Mitomycin C Against Multidrug-Resistant Gram-Negative Bacteria.* FRONTIERS IN MICROBIOLOGY, 2019. **10**.

2. She, P., et al., *Repurposing 9-Aminoacridine as an Adjuvant Enhances the Antimicrobial Effects of Rifampin against Multidrug-Resistant Klebsiella pneumoniae.* Microbiol Spectr, 2023. **11**(3): p. e0447422.

3. Shailaja, S., et al., *Identification of a potential inhibitor for New Delhi metallo-β-lactamase 1 (NDM-1) from FDA approved chemical library- a drug repurposing approach to combat carbapenem resistance.* J Biomol Struct Dyn, 2023. **41**(16): p. 7700-7711.

4. Abdelaziz, A.A., et al., *Unveiling the antibacterial action of ambroxol against Staphylococcus aureus bacteria: in vitro, in vivo, and in silico investigation.* BMC Microbiol, 2024. **24**(1): p. 507.

5. De S. Machado, C., et al., *In vitro evaluation of the antibacterial activity of amitriptyline and its synergistic effect with ciprofloxacin, sulfamethoxazole-trimethoprim, and colistin as an alternative in drug repositioning.* MEDICINAL CHEMISTRY RESEARCH, 2020. **29**(1): p. 166-177.

6. Sharma, P., et al., *Antimicrobial Proficiency of Amlodipine: Investigating its Impact on Pseudomonas spp. in Urinary Tract Infections.* INDIAN JOURNAL OF MICROBIOLOGY, 2024.

7. Andrade, M., et al., *Evaluation of Amlodipine and Imipramine Efficacy to Treat Galleria mellonella Infection by Biofilm-Producing and Antimicrobial-Resistant Staphylococcus aureus.* ANTIBIOTICS-BASEL, 2025. **14**(2).

8. Foletto, V.S., et al., *Repositioning of antidepressant drugs and synergistic effect with ciprofloxacin against multidrug-resistant bacteria.* World J Microbiol Biotechnol, 2021. **37**(3): p. 53.

9. Cao, D., et al., *Repurposing astemizole to kill multidrug-resistant bacteria isolated in general surgery.* Microb Pathog, 2025. **200**: p. 107369.

10. Kornelsen, V., M. Unger, and A. Kumar, *Atorvastatin does not display an antimicrobial activity on its own nor potentiates the activity of other antibiotics against Acinetobacter baumannii ATCC17978 or A. baumannii AB030.* Access microbiology, 2021. **3**(11): p. 000288-000288.

11. Aguinagalde, L., et al., *Auranofin efficacy against MDR Streptococcus pneumoniae and Staphylococcus aureus infections.* JOURNAL OF ANTIMICROBIAL CHEMOTHERAPY, 2015. **70**(9): p. 2608-2617.

12. Thangamani, S., et al., *Repurposing auranofin for the treatment of cutaneous staphylococcal infections.* Int J Antimicrob Agents, 2016. **47**(3): p. 195-201.

13. She, P., et al., *Synergistic Microbicidal Effect of Auranofin and Antibiotics Against Planktonic and Biofilm-Encased S. aureus and E. faecalis.* FRONTIERS IN MICROBIOLOGY, 2019. **10**.

14. She, P., et al., *Antibiofilm efficacy of the gold compound auranofin on dual species biofilms of Staphylococcus aureus and Candida sp.* J Appl Microbiol, 2020. **128**(1): p. 88-101.

15. Mohammad, H., N.S. Abutaleb, and M.N. Seleem, *Auranofin Rapidly Eradicates Methicillin-resistant Staphylococcus aureus (MRSA) in an Infected Pressure Ulcer Mouse Model.* Sci Rep, 2020. **10**(1).

16. Feng, X., et al., *Synergistic Activity of Colistin Combined With Auranofin Against Colistin-Resistant Gram-Negative Bacteria.* FRONTIERS IN MICROBIOLOGY, 2021. **12**.

17. Ding, X., et al., *A Macromolecule Reversing Antibiotic Resistance Phenotype and Repurposing Drugs as Potent Antibiotics.* ADVANCED SCIENCE, 2020. **7**(17).

18. Sun, H., et al., *Resensitizing carbapenem- and colistin-resistant bacteria to antibiotics using auranofin.* Nat Commun, 2020. **11**(1).

19. Chen, H., et al., *Synergistic Microbicidal Effect of AUR and PEITC Against Staphylococcus aureus Skin Infection.* Front Cell Infect Microbiol, 2022. **12**.

20. Yu, Y., et al., *Repurposing Non-Antibiotic Drugs Auranofin and Pentamidine in Combination to Combat Multidrug-Resistant Gram-Negative Bacteria.* Int J Antimicrob Agents, 2022. **59**(5): p. 106582.

21. Kim, H.-R. and Y.-B. Eom, *Auranofin promotes antibacterial effect of doripenem against carbapenem-resistant Acinetobacter baumannii.* J Appl Microbiol, 2022. **133**(3): p. 1422-1433.

22. Meng, Q., et al., *Repurposing Benzbromarone as an Antibacterial Agent against Gram-Positive Bacteria.* ACS Infect Dis, 2024. **10**(12): p. 4208-4221.

23. da Rosa, T.F., et al., *Repositioning of Benzodiazepine Drugs and Synergistic Effect with Ciprofloxacin Against ESKAPE Pathogens.* Curr Microbiol, 2023. **80**(5).

24. Liu, Y., et al., *Reversion of antibiotic resistance in multidrug-resistant pathogens using non-antibiotic pharmaceutical benzydamine.* COMMUNICATIONS BIOLOGY, 2021. **4**(1).

25. She, P., et al., *Drug Repurposing: In vitro and in vivo Antimicrobial and Antibiofilm Effects of Bithionol Against Enterococcus faecalis and Enterococcus faecium.* FRONTIERS IN MICROBIOLOGY, 2021. **12**.

26. Zhang, N., et al., *Repurposing the Hedgehog pathway inhibitor, BMS-833923, as a phosphatidylglycerol-selective membrane-disruptive colistin adjuvant against ESKAPE pathogens.* Int J Antimicrob Agents, 2023. **62**(3).

27. Palaniappan, B., A.P. Solomon, and D.C. Raj, *Targeting AgrA quorum sensing regulator by bumetanide attenuates virulence in Staphylococcus aureus - A drug repurposing approach.* LIFE SCIENCES, 2021. **273**.

28. Chen, C., et al., *Antibacterial Activity and Mechanism of Candesartan Cilexetil against Enterococcus faecalis.* ACS OMEGA, 2024. **9**(19): p. 21510-21519.

29. Xu, L., et al., *Repurposing Candesartan Cilexetil as Antibacterial Agent for MRSA Infection.* FRONTIERS IN MICROBIOLOGY, 2021. **12**.

30. Li, H., et al., *Caspofungin enhances the potency of rifampin against Gram-negative bacteria.* FRONTIERS IN MICROBIOLOGY, 2024. **15**.

31. Thangamani, S., W. Younis, and M.N. Seleem, *Repurposing celecoxib as a topical antimicrobial agent.* FRONTIERS IN MICROBIOLOGY, 2015. **6**.

32. Liu, S., et al., *Insights into the antimicrobial effects of ceritinib against Staphylococcus aureus in vitro and in vivo by cell membrane disruption.* AMB Express, 2022. **12**(1).

33. Xu, C., et al., *Repurposing cetylpyridinium chloride and domiphen bromide as phosphoethanolamine transferase inhibitor to combat colistin-resistant Enterobacterales.* Microbiol Res, 2024. **288**.

34. Carlson-Banning, K.M., et al., *Toward repurposing ciclopirox as an antibiotic against drug-resistant Acinetobacter baumannii, Escherichia coli, and Klebsiella pneumoniae.* PLoS One, 2013. **8**(7): p. e69646.

35. Zakaria, A.S., E.A. Edward, and N.M. Mohamed, *Evaluation of Ciclopirox as a Virulence-modifying Agent Against Multidrug Resistant Pseudomonas aeruginosa Clinical Isolates from Egypt.* Microbiology and Biotechnology Letters, 2019. **47**(4): p. 651-661.

36. Cho, H. and K.-S. Kim, *Repurposing of Ciclopirox to Overcome the Limitations of Zidovudine (Azidothymidine) against Multidrug-Resistant Gram-Negative Bacteria.* PHARMACEUTICS, 2022. **14**(3).

37. Fang, Z.-Y., et al., *Repurposing cinacalcet suppresses multidrug-resistant Staphylococcus aureus by disruption of cell membrane and inhibits biofilm by targeting IcaR.* JOURNAL OF ANTIMICROBIAL CHEMOTHERAPY, 2024. **79**(4): p. 903-917.

38. Tran, T.B., et al., *Anthelmintic closantel enhances bacterial killing of polymyxin B against multidrug-resistant Acinetobacter baumannii.* JOURNAL OF ANTIBIOTICS, 2016. **69**(6): p. 415-421.

39. Ding, T., et al., *Synergistic antibacterial effects of closantel and its enantiomers in combination with colistin against multidrug resistant gram-negative bacteria.* FRONTIERS IN MICROBIOLOGY, 2024. **15**.

40. Zheng, Y.-D., et al., *Crizotinib Shows Antibacterial Activity against Gram-Positive Bacteria by Reducing ATP Production and Targeting the CTP Synthase PyrG.* Microbiol Spectr, 2022. **10**(3).

41. Eduvirgem, J., et al., *Antimicrobial and antibiofilm activities of desloratadine against multidrug-resistant Acinetobacter baumannii.* Future Microbiol, 2023. **18**: p. 15-25.

42. Abbas, H.A., et al., *Diclofenac mitigates virulence of multidrug-resistantStaphylococcus aureus.* Arch Microbiol, 2020. **202**(10): p. 2751-2760.

43. Yuan, Y., et al., *Repurposing Dimetridazole and Ribavirin to disarm Pseudomonas aeruginosa virulence by targeting the quorum sensing system.* FRONTIERS IN MICROBIOLOGY, 2022. **13**.

44. Rios, T.B., et al., *Repurposing streptomycin and chloramphenicol against bacterial pathogens by combination with diminazene aceturate.* Letters in Applied Microbiology, 2023. **76**(1).

45. Thakare, R., et al., *Repurposing disulfiram for treatment of Staphylococcus aureus infections.* Int J Antimicrob Agents, 2019. **53**(6): p. 709-715.

46. Serafin, M.B., et al., *Repositioning of Disulfiram in Association with Vancomycin Against Enterococcus spp. MDR and XDR.* Curr Microbiol, 2022. **79**(5).

47. Elfaky, M.A.A., et al., *Drug repositioning: doxazosin attenuates the virulence factors and biofilm formation in Gram-negative bacteria.* Appl Microbiol Biotechnol, 2023. **107**(11): p. 3763-3778.

48. Zhang, L., et al., *Doxifluridine effectively kills antibiotic-resistant Staphylococcus aureus in chronic obstructive pulmonary disease.* Microbiol Spectr, 2024. **12**(12).

49. Thangamani, S., W. Younis, and M.N. Seleem, *Repurposing Clinical Molecule Ebselen to Combat Drug Resistant Pathogens.* PLoS One, 2015. **10**(7).

50. Thangamani, S., W. Younis, and M.N. Seleem, *Repurposing ebselen for treatment of multidrug-resistant staphylococcal infections.* Sci Rep, 2015. **5**: p. 11596.

51. AbdelKhalek, A., et al., *Repurposing ebselen for decolonization of vancomycin-resistant enterococci (VRE).* PLoS One, 2018. **13**(6).

52. Lee, H., et al., *Repurposing Eltrombopag for Multidrug Resistant Staphylococcus aureus Infections.* ANTIBIOTICS-BASEL, 2021. **10**(11).

53. Henry, S.A., et al., *Steroid Drugs Inhibit Bacterial Respiratory Oxidases and Are Lethal Toward Methicillin-Resistant Staphylococcus aureus.* JOURNAL OF INFECTIOUS DISEASES, 2024. **230**(1): p. e149-e158.

54. Kumar, A., et al., *Repurposing ethyl bromopyruvate as a broad-spectrum antibacterial.* JOURNAL OF ANTIMICROBIAL CHEMOTHERAPY, 2019. **74**(4): p. 912-920.

55. Pi, H., et al., *Repurposing of the Fasciolicide Triclabendazole to Treat Infections Caused by Staphylococcus spp. and Vancomycin-Resistant Enterococci.* MICROORGANISMS, 2021. **9**(8).

56. Ahmed, S.A., et al., *Potential Synergy of Fluoxetine and Antibacterial Agents Against Skin and Soft Tissue Pathogens and Drug-Resistant Organisms.* ANTIBIOTICS-BASEL, 2024. **13**(12).

57. Seleem, N.M., et al., *Drugs with new lease of life as quorum sensing inhibitors: for combating MDR Acinetobacter baumannii infections.* EUROPEAN JOURNAL OF CLINICAL MICROBIOLOGY & INFECTIOUS DISEASES, 2020. **39**(9): p. 1687-1702.

58. Kaul, M., et al., *Combination with a FtsZ inhibitor potentiates the in vivo efficacy of oxacillin against methicillin-resistant Staphylococcus aureus.* MEDICINAL CHEMISTRY RESEARCH, 2022. **31**(10): p. 1705-1715.

59. Wu, Y., et al., *Repurposed Anti-Multiple Sclerosis Drug Fty720 Targets Carbapenem-Resistant Acinetobacter baumannii via Multiple Pathways.* Curr Microbiol, 2025. **82**(1).

60. Jordheim, L.P., et al., *Gemcitabine is active against clinical multiresistant Staphylococcus aureus strains and is synergistic with gentamicin.* Int J Antimicrob Agents, 2012. **39**(5): p. 444-447.

61. Farha, A.K., O. Habimana, and H. Corke, *Guanabenz acetate, an antihypertensive drug repurposed as an inhibitor of Escherichia coli biofilm.* Microbiol Spectr, 2024. **12**(11).

62. Liu, S., et al., *Antibacterial and antibiofilm efficacy of repurposing drug hexestrol against methicillin-resistant Staphylococcus aureus.* International Journal of Medical Microbiology, 2023. **313**(2).

63. Stefany Aires do Nascimento, F.B., et al., *Antimicrobial activity of hydralazine against methicillin-resistant and methicillin-susceptible Staphylococcus aureus.* Future Microbiol, 2024. **19**(2): p. 91-106.

64. Manzanelli, F.A., et al., *Sodium ibuprofenate: antibacterial activities and potential β-lactamase inhibition in critical Gram-negative bacteria.* Future Microbiol, 2025.

65. Thakare, R., et al., *Repurposing Ivacaftor for treatment of Staphylococcus aureus infections.* Int J Antimicrob Agents, 2017. **50**(3): p. 389-392.

66. Leitao, A.C., et al., *Antibacterial activity of menadione alone and in combination with oxacillin against methicillin-resistant Staphylococcus aureus and its impact on biofilms.* J Med Microbiol, 2023. **72**(9).

67. Muteeb, G., et al., *Risedronate and Methotrexate Are High-Affinity Inhibitors of New Delhi Metallo-β-Lactamase-1 (NDM-1): A Drug Repurposing Approach.* Molecules, 2022. **27**(4).

68. Cruz-Muñiz, M.Y., et al., *Repurposing the anticancer drug mitomycin C for the treatment of persistent Acinetobacter baumannii infections.* Int J Antimicrob Agents, 2017. **49**(1): p. 88-92.

69. Svedholm, E., et al., *Repurposing Mitomycin C in Combination with Pentamidine or Gentamicin to Treat Infections with Multi-Drug-Resistant (MDR) Pseudomonas aeruginosa.* ANTIBIOTICS-BASEL, 2024. **13**(2).

70. Xu, C., et al., *Imidazole Type Antifungal Drugs Are Effective Colistin Adjuvants That Resensitize Colistin-ResistantEnterobacteriaceae.* Advanced Therapeutics, 2020. **3**(9).

71. Mahey, N., et al., *Antifungal Azoles as Tetracycline Resistance Modifiers in Staphylococcus aureus.* APPLIED AND ENVIRONMENTAL MICROBIOLOGY, 2021. **87**(15).

72. Torres, N.S., et al., *Screening a Commercial Library of Pharmacologically Active Small Molecules against Staphylococcus aureus Biofilms.* Antimicrob Agents Chemother, 2016. **60**(10): p. 5663-5672.

73. Sun, W., et al., *Rapid antimicrobial susceptibility test for identification of new therapeutics and drug combinations against multidrug-resistant bacteria.* EMERGING MICROBES & INFECTIONS, 2016. **5**.

74. Peyclit, L., et al., *In Vitro Screening of a 1280 FDA-Approved Drugs Library against Multidrug-Resistant and Extensively Drug-Resistant Bacteria.* ANTIBIOTICS-BASEL, 2022. **11**(3).

75. Van den Driessche, F., et al., *Screening a repurposing library for potentiators of antibiotics against Staphylococcus aureus biofilms.* Int J Antimicrob Agents, 2017. **49**(3): p. 315-320.

76. Trombetta, R.P., et al., *A High-Throughput Screening Approach To Repurpose FDA-Approved Drugs for Bactericidal Applications against Staphylococcus aureus Small-Colony Variants.* MSPHERE, 2018. **3**(5).

77. Domalaon, R., et al., *Synergistic combinations of anthelmintic salicylanilides oxyclozanide, rafoxanide, and closantel with colistin eradicates multidrug-resistant colistin-resistant Gram-negative bacilli.* J Antibiot (Tokyo), 2019. **72**(8): p. 605-616.

78. Cheng, Y.S., et al., *Repurposing Screen Identifies Unconventional Drugs With Activity Against Multidrug Resistant Acinetobacter baumannii.* Front Cell Infect Microbiol, 2018. **8**: p. 438.

79. Aguilar-Vega, L., et al., *Antibacterial properties of phenothiazine derivatives against multidrug-resistant Acinetobacter baumannii strains.* J Appl Microbiol, 2021. **131**(5): p. 2235-2243.

80. Mahey, N., et al., *Repurposing Approved Drugs as Fluoroquinolone Potentiators to Overcome Efflux Pump Resistance in Staphylococcus aureus.* Microbiol Spectr, 2021. **9**(3): p. e0095121.

81. Gargvanshi, S. and W.G. Gutheil, *Library Screening for Synergistic Combinations of FDA-Approved Drugs and Metabolites with Vancomycin against VanA-Type Vancomycin-Resistant Enterococcus faecium.* Microbiol Spectr, 2022. **10**(5): p. e0141222.

82. Di Bonaventura, G., et al., *Repurposing High-Throughput Screening Identifies Unconventional Drugs with Antibacterial and Antibiofilm Activities against Pseudomonas aeruginosa under Experimental Conditions Relevant to Cystic Fibrosis.* Microbiol Spectr, 2023. **11**(4).

83. Ugurel, E. and D. Turgut-Balik, *Synergistic combination of carvedilol, amlodipine, amitriptyline, and antibiotics as an alternative treatment approach for the susceptible and multidrug-resistant A. baumannii infections via drug repurposing.* Eur J Clin Microbiol Infect Dis, 2023. **42**(9): p. 1063-1072.

84. Carmona-Orozco, M.L. and F. Echeverri, *Induction of biofilm in extended-spectrum beta-lactamase Staphylococcus aureus with drugs commonly used in pharmacotherapy.* Microb Pathog, 2024. **195**.

85. Salvaterra Pasquini, J.P., et al., *Polymyxin B adjuvants against polymyxin B- and carbapenem-resistant Gram-negative bacteria.* Future Microbiol, 2024. **19**(17): p. 1445-1454.

86. de Melo Guedes, G.M., et al., *Repurposing approved drugs as potential efflux pump inhibitors in multidrug-resistant Pseudomonas aeruginosa.* Future Microbiol, 2024. **19**(6): p. 495-508.

87. Sharma, P., et al., *Repurposing drugs as uS12 ribosomal protein inhibitors to overcome UTI resistance in MDR Pseudomonas strains: In silico and in vitro study.* INDIAN JOURNAL OF BIOCHEMISTRY & BIOPHYSICS, 2024. **61**(11): p. 672-687.

88. Das, S., et al., *Transcriptomics and systems biology identify non-antibiotic drugs for the treatment of ocular bacterial infection.* iScience, 2022. **25**(9).

89. Jiao, F., et al., *Identification of Potential PBP2a Inhibitors Against Methicillin-Resistant Staphylococcus aureus via Drug Repurposing and Combination Therapy.* CHEMICAL BIOLOGY & DRUG DESIGN, 2025. **105**(3).

90. Lau, Q.Y., et al., *An FDA-Drug Library Screen for Compounds with Bioactivities against Meticillin-Resistant Staphylococcus aureus (MRSA).* Antibiotics (Basel, Switzerland), 2015. **4**(4): p. 424-34.

91. Domalaon, R., et al., *The Anthelmintic Drug Niclosamide Synergizes with Colistin and Reverses Colistin Resistance in Gram-Negative Bacilli.* Antimicrob Agents Chemother, 2019. **63**(4).

92. Rajamuthiah, R., et al., *Repurposing salicylanilide anthelmintic drugs to combat drug resistant Staphylococcus aureus.* PLoS One, 2015. **10**(4): p. e0124595.

93. Mohammad, H., et al., *Repurposing niclosamide for intestinal decolonization of vancomycin-resistant enterococci.* Int J Antimicrob Agents, 2018. **51**(6): p. 897-904.

94. Pacios, O., et al., *Adaptation of clinical isolates of Klebsiella pneumoniae to the combination of niclosamide with the efflux pump inhibitor phenyl-arginine-β-naphthylamide (PaβN): co-resistance to antimicrobials.* JOURNAL OF ANTIMICROBIAL CHEMOTHERAPY, 2022. **77**(5): p. 1272-1281.

95. Lu, T., et al., *Novel niclosamide-derived adjuvants elevating the efficacy of polymyxin B against MDR Pseudomonas aeruginosa DK2.* Eur J Med Chem, 2022. **236**: p. 114318.

96. Zhang, W., et al., *Niclosamide as a repurposing drug against Gram-positive bacterial infections.* JOURNAL OF ANTIMICROBIAL CHEMOTHERAPY, 2022. **77**(12): p. 3312-3320.

97. Kaul, G., et al., *Nitazoxanide potentiates linezolid against linezolid-resistant Staphylococcus aureus in vitro and in vivo.* JOURNAL OF ANTIMICROBIAL CHEMOTHERAPY, 2022. **77**(9): p. 2456-2460.

98. Xu, M., et al., *Antiparasitic nitazoxanide potentiates colistin against colistin-resistant Acinetobacter baumannii and Escherichia coli in vitro and in vivo.* Microbiol Spectr, 2024. **12**(1): p. e0229523.

99. Jiang, X., et al., *Nitazoxanide synergizes polymyxin B against Escherichia coli by depleting cellular energy.* Microbiol Spectr, 2024. **12**(8).

100. Bakht, P., et al., *Repurposing of non-steroidal anti-inflammatory drugs for combination therapies to combat multidrug-resistant S. aureus of bovine reproductive tract origin.* Vet Res Commun, 2024. **48**(3): p. 1497-1510.

101. Foletto, V.S., et al., *Repositioning of fluoxetine and paroxetine: study of potential antibacterial activity and its combination with ciprofloxacin.* MEDICINAL CHEMISTRY RESEARCH, 2020. **29**(3): p. 556-563.

102. Zhou, L., et al., *Repurposing Antispasmodic Agent Otilonium Bromide for Treatment ofStaphylococcus aureusInfections.* FRONTIERS IN MICROBIOLOGY, 2020. **11**.

103. Kaul, G., et al., *Oxiconazole Potentiates Gentamicin against Gentamicin-Resistant Staphylococcus aureus In Vitro and In Vivo.* Microbiol Spectr, 2023. **11**(4): p. e0503122.

104. Ayerbe-Algaba, R., et al., *The anthelmintic oxyclozanide restores the activity of colistin against colistin-resistant Gram-negative bacilli.* Int J Antimicrob Agents, 2019. **54**(4): p. 507-512.

105. Cabral, V.P.F., et al., *Antibacterial activity of paroxetine against Staphylococcus aureus and possible mechanisms of action.* Future Microbiol, 2023. **18**(7): p. 415-426.

106. Liu, Y., et al., *Antimicrobial, Antibiofilm, and Anti-persister Activities of Penfluridol Against Staphylococcus aureus.* FRONTIERS IN MICROBIOLOGY, 2021. **12**.

107. Wu, C., et al., *Pentamidine sensitizes FDA-approved non-antibiotics for the inhibition of multidrug-resistant Gram-negative pathogens.* Eur J Clin Microbiol Infect Dis, 2020. **39**(9): p. 1771-1779.

108. Zhang, Y., et al., *Synergistic Effect of Colistin Combined with PFK-158 against Colistin-Resistant Enterobacteriaceae.* Antimicrob Agents Chemother, 2019. **63**(7).

109. Ito, R., et al., *Inhibition of Fosfomycin Resistance Protein FosA by Phosphonoformate (Foscarnet) in Multidrug-Resistant Gram-Negative Pathogens.* Antimicrob Agents Chemother, 2017. **61**(12).

110. Kumar, S., et al., *Antimicrobial effect of pimozide by targeting ROS-mediated killing in Staphylococcus aureus.* Biotechnol Appl Biochem, 2023. **70**(5): p. 1679-1689.

111. Guedes, R.F.d.M., et al., *Antimicrobial and antibiofilm effect of promethazine on bacterial isolates from canine otitis externa: an in vitro study.* Microb Pathog, 2024. **196**.

112. Bendary, M.M., et al., *Therapeutic Switching of Rafoxanide: a New Approach To Fighting Drug-Resistant Bacteria and Fungi.* Microbiol Spectr, 2023. **11**(4): p. e0267922.

113. Ferreira, M., et al., *Rifabutin: a repurposed antibiotic with high potential against planktonic and biofilm staphylococcal clinical isolates.* FRONTIERS IN MICROBIOLOGY, 2024. **15**.

114. Tantisuwanno, C., et al., *Synergism between Rifampicin and Cationic Polyurethanes Overcomes Intrinsic Resistance of Escherichia coli.* BIOMACROMOLECULES, 2021. **22**(7): p. 2910-2920.

115. She, P., et al., *Pixantrone Sensitizes Gram-Negative Pathogens to Rifampin.* Microbiol Spectr, 2022. **10**(6).

116. Chromy, B.A., et al., *Repurposing screens identify rifamycins as potential broad-spectrum therapy for multidrug-resistant Acinetobacter baumannii and select agent microorganisms.* Future Microbiol, 2012. **7**(8): p. 1011-1020.

117. Wang, C., et al., *Discovery of Salifungin as a Repurposed Antibiotic against Methicillin-Resistant Staphylococcus aureus with Limited Resistance Development.* ACS Infect Dis, 2024. **10**(5): p. 1576-1589.

118. Folliero, V., et al., *Repurposing Selamectin as an Antimicrobial Drug against Hospital-Acquired Staphylococcus aureus Infections.* MICROORGANISMS, 2023. **11**(9).

119. Hussein, M.H., et al., *From Breast Cancer to Antimicrobial: Combating Extremely Resistant Gram-Negative "Superbugs" Using Novel Combinations of Polymyxin B with Selective Estrogen Receptor Modulators.* Microb Drug Resist, 2017. **23**(5): p. 640-650.

120. Serafin, M.B., et al., *Synergistic effect of sertraline and disulfiram against multidrug resistant bacteria as a new alternative to drug repositioning.* BRAZILIAN JOURNAL OF PHARMACEUTICAL SCIENCES, 2020. **56**.

121. Endo, T.H., et al., *Selective Serotonin Reuptake Inhibitors: Antimicrobial Activity Against ESKAPEE Bacteria and Mechanisms of Action.* ANTIBIOTICS-BASEL, 2025. **14**(1).

122. Barin, T.d.M., et al., *Antimicrobial, Synergistic, and Antibiofilm Activity of Sildenafil Against Pseudomonas aeruginosa: Preliminary Studies.* APPLIED SCIENCES-BASEL, 2024. **14**(22).

123. Li, Y., et al., *Anti-hepatitis C virus drug simeprevir: a promising antimicrobial agent against MRSA.* Appl Microbiol Biotechnol, 2022. **106**(7): p. 2689-2702.

124. Wu, Y., et al., *Simeprevir restores the anti-Staphylococcus activity of polymyxins.* AMB Express, 2023. **13**(1).

125. Graziano, T.S., et al., *Statins and Antimicrobial Effects: Simvastatin as a Potential Drug against Staphylococcus aureus Biofilm.* PLoS One, 2015. **10**(5).

126. Thangamani, S., et al., *Exploring simvastatin, an antihyperlipidemic drug, as a potential topical antibacterial agent.* Sci Rep, 2015. **5**.

127. Moglad, E., et al., *Repurposing simvastatin for treatment of Klebsiella pneumoniae infections: in vitro and in vivo study.* BIOFOULING, 2024. **40**(10): p. 801-815.

128. Cortes, I.T., K.d.P. Silva, and K. Cogo-Mueller, *Effects of simvastatin on the mevalonate pathway and cell wall integrity of Staphylococcus aureus.* J Appl Microbiol, 2025. **136**(1).

129. She, P., et al., *Repurposing of the antimalarial agent tafenoquine to combat MRSA.* MSYSTEMS, 2023. **8**(6).

130. Herrera-Espejo, S., et al., *Efficacy of Tamoxifen Metabolites in Combination with Colistin and Tigecycline in Experimental Murine Models of Escherichia coli and Acinetobacter baumannii.* ANTIBIOTICS-BASEL, 2024. **13**(5).

131. Liu, S., et al., *Drug synergy discovery of tavaborole and aminoglycosides against Escherichia coli using high throughput screening.* AMB Express, 2022. **12**(1).

132. Maiden, M.M., et al., *Triclosan Is an Aminoglycoside Adjuvant for Eradication of Pseudomonas aeruginosa Biofilms.* Antimicrob Agents Chemother, 2018. **62**(6).

133. Younis, W., S. Thangamani, and M.N. Seleem, *Repurposing Non-Antimicrobial Drugs and Clinical Molecules to Treat Bacterial Infections.* Curr Pharm Des, 2015. **21**(28): p. 4106-11.

134. Long, T.E., *Repurposing Thiram and Disulfiram as Antibacterial Agents for Multidrug-Resistant Staphylococcus aureus Infections.* Antimicrob Agents Chemother, 2017. **61**(9).

135. Wu, R., et al., *Bactericidal and anti-quorum sensing activity of repurposing drug Visomitin against Staphylococcus aureus.* VIRULENCE, 2024. **15**(1).

136. Ng, S.M.S., et al., *Repurposing Zidovudine in combination with Tigecycline for treating carbapenem-resistant Enterobacteriaceae infections.* Eur J Clin Microbiol Infect Dis, 2018. **37**(1): p. 141-148.

137. Falagas, M.E., et al., *Synergistic activity of colistin with azidothymidine against colistin-resistant Klebsiella pneumoniae clinical isolates collected from inpatients in Greek hospitals.* Int J Antimicrob Agents, 2019. **53**(6): p. 855-858.

138. Hu, Y., Y. Liu, and A. Coates, *Azidothymidine Produces Synergistic Activity in Combination with Colistin against Antibiotic-Resistant Enterobacteriaceae.* Antimicrob Agents Chemother, 2019. **63**(1).

139. DeSarno, A.E., B.J. Parcell, and P.J. Coote, *Repurposing the anti-viral drug zidovudine (AZT) in combination with meropenem as an effective treatment for infections with multi-drug resistant, carbapenemase-producing strains of Klebsiella pneumoniae.* Pathog Dis, 2020. **78**(9).

140. Gómara-Lomero, M., et al., *Zidovudine multi-combos with last-line fosfomycin, ceftazidime-avibactam, colistin and tigecycline against Multi-Drug Resistant Klebsiella pneumoniae*. 2022.

141. Antonello, R.M., et al., *Zidovudine in synergistic combination with fosfomycin: an in vitro and in vivo evaluation against multidrug-resistant Enterobacterales.* Int J Antimicrob Agents, 2021. **58**(1): p. 106362.
